# TORC1 controls G_1_–S cell cycle transition in yeast via Mpk1 and the greatwall kinase pathway

**DOI:** 10.1038/ncomms9256

**Published:** 2015-09-10

**Authors:** Marta Moreno-Torres, Malika Jaquenoud, Claudio De Virgilio

**Affiliations:** 1 Department of Biology, University of Fribourg, Chemin du Musée 10, Fribourg CH-1700, Switzerland

## Abstract

The target of rapamycin complex 1 (TORC1) pathway couples nutrient, energy and hormonal signals with eukaryotic cell growth and division. In yeast, TORC1 coordinates growth with G_1_–S cell cycle progression, also coined as START, by favouring the expression of G_1_ cyclins that activate cyclin-dependent protein kinases (CDKs) and by destabilizing the CDK inhibitor Sic1. Following TORC1 downregulation by rapamycin treatment or nutrient limitation, clearance of G_1_ cyclins and C-terminal phosphorylation of Sic1 by unknown protein kinases are both required for Sic1 to escape ubiquitin-dependent proteolysis prompted by its flagging via the SCF^Cdc4^ (Skp1/Cul1/F-box protein) ubiquitin ligase complex. Here we show that the stabilizing phosphorylation event within the C-terminus of Sic1 requires stimulation of the mitogen-activated protein kinase, Mpk1, and inhibition of the Cdc55 protein phosphatase 2A (PP2A^Cdc55^) by greatwall kinase-activated endosulfines. Thus, Mpk1 and the greatwall kinase pathway serve TORC1 to coordinate the phosphorylation status of Sic1 and consequently START with nutrient availability.

## Abstract (web summary)

The target of rapamycin complex 1 (TORC1) pathway couples nutrient availability with cell growth and division by destabilizing the cyclin-dependent kinase (CDK) inhibitor Sic1. Here the authors show that TORC1 downregulation leads to stabilization of Sic1 via phosphorylation by the MAP kinase Mpk1 and inhibition of dephosphorylation via the greatwall kinase pathway.

## Graphical abstract

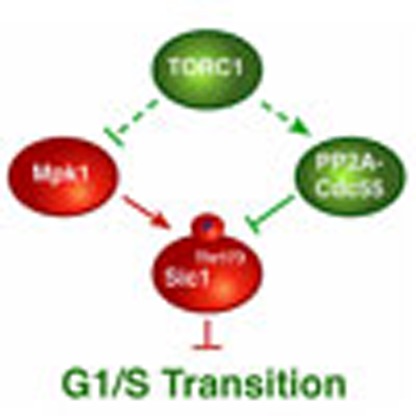

Nutrient signalling drives protein kinase activity of target of rapamycin complex 1 (TORC1) to stimulate anabolic, growth-related processes (for example, protein biosynthesis) in concert with cell cycle transition events^1^,^2^. TORC1 has primarily been appreciated for its role in coordinating growth with the G_1_–S cell cycle transition, or START in yeast^3^, but recent data indicate that TORC1 also contributes to the fine-tuning of other cell cycle events (for example, G_2_–M transition) to environmental cues^4^,^5^. TORC1 favours the G_1_–S transition in part by promoting transcription and translation of the cell cycle regulatory G_1_ cyclins^4^,^6^,^7^,^8^. However, detailed mechanistic insight into TORC1-regulated G_1_ cyclin expression is still sporadic and incomplete. A well-studied example in yeast indicates that the Cln3 G_1_ cyclin levels, and consequently START-promoting G_1_ cyclin-dependent protein kinase (CDK; Cln-Cdc28) activity, are specifically sustained by TORC1-mediated stimulation of translation initiation. The latter is required for ribosomes to bypass a translational repressive upstream open reading frame and reach the start codon of the 5′-untranslated region within the *CLN3* messenger RNA (mRNA)^7^,^9^. In parallel to favouring G_1_ cyclin expression, TORC1 further couples cell growth with cell cycle progression by antagonizing the expression and/or function of CDK inhibitors (CDKIs) that restrain CDK-mediated G_1_–S transition^4^. Although the underlying mechanistic details remain poorly understood, progress has also been made in this area. An example in yeast, again, is the CDKI Sic1, which binds, following G_1_ CDK-dependent multi-site phosphorylation, the F-box protein Cdc4 of the SCF^Cdc4^ ubiquitin ligase complex that flags it for ubiquitin-dependent proteolysis^10^,^11^,^12^,^13^. TORC1 apparently triggers Sic1 degradation not only by ensuring G_1_ CDK activation but also by confining the phosphorylation of specific residue(s) (for example, Thr^173^) in Sic1 (ref. 6). The details of the latter regulatory mechanism, however, are still elusive.

Attenuation of signalling through TORC1 (for example, following carbon and/or nitrogen limitation) incites yeast cells to arrest in G_1_ of the cell cycle and enter a quiescent state that is characterized by a distinct array of physiological, biochemical and morphological traits^14^,^15^. The protein kinase Rim15 orchestrates quiescence (including proper G_1_ arrest) when released from inhibition by the AGC family kinase, Sch9, which requires, analogously to mammalian S6 kinase (S6K), activation by TORC1 (refs 16, 17, 18, 19). Like the orthologous greatwall kinases (Gwl) in higher eukaryotes, Rim15 controls some of its distal readouts by phosphorylating a conserved residue within endosulfines (that is, Igo1/2 in yeast), thereby converting them to inhibitors of the Cdc55 protein phosphatase 2A (PP2A^Cdc55^; or PP2A-B55 in higher eukaryotes)^20^,^21^,^22^. The Gwl signalling branch in yeast (Rim15-Igo1/2-PP2A^Cdc55^) mediates the activation of a quiescence-specific gene expression programme in part via the transcriptional activator Gis1 and likely additional factors that protect specific mRNAs from degradation via the 5′–3′ mRNA decay pathway^22^,^23^,^24^,^25^. Whether Rim15 also controls cell cycle arrest in G_1_ via Igo1/2-PP2A^Cdc55^ is currently not known. Interestingly, in this context, *Xenopus*, *Drosophila* and likely human cells employ their respective greatwall kinase pathway (Gwl-endosulfine-B55) to maintain high-level phosphorylation of cyclin B-CDK1 substrates, thereby promoting mitotic entry^26^,^27^. In yeast, however, the Gwl signalling branch contributes only marginally to the regulation of mitotic entry^28^,^29^, likely because TORC1 curtails signalling through Rim15 in exponentially growing cells.

Here we show that TORC1 inhibition and consequently activation of Igo1/2 by the Gwl Rim15 serves to antagonize PP2A^Cdc55^ and prevent it from dephosphorylating pThr^173^ within the CDKI Sic1. This specific phosphorylation event depends on the mitogen-activated protein kinase (MAPK) Mpk1 and ensures protection of Sic1 from SCF^Cdc4^-mediated ubiquitination and subsequent proteolysis to enable it to grant proper G_1_ arrest when TORC1 is downregulated. Thus, TORC1 coordinates the phosphorylation status of Sic1 and consequently G_1_–S cell cycle progression with nutrient availability via Mpk1 and the greatwall kinase pathway.

## Results

### The greatwall kinase pathway controls Sic1 stability

**Figure 1:**
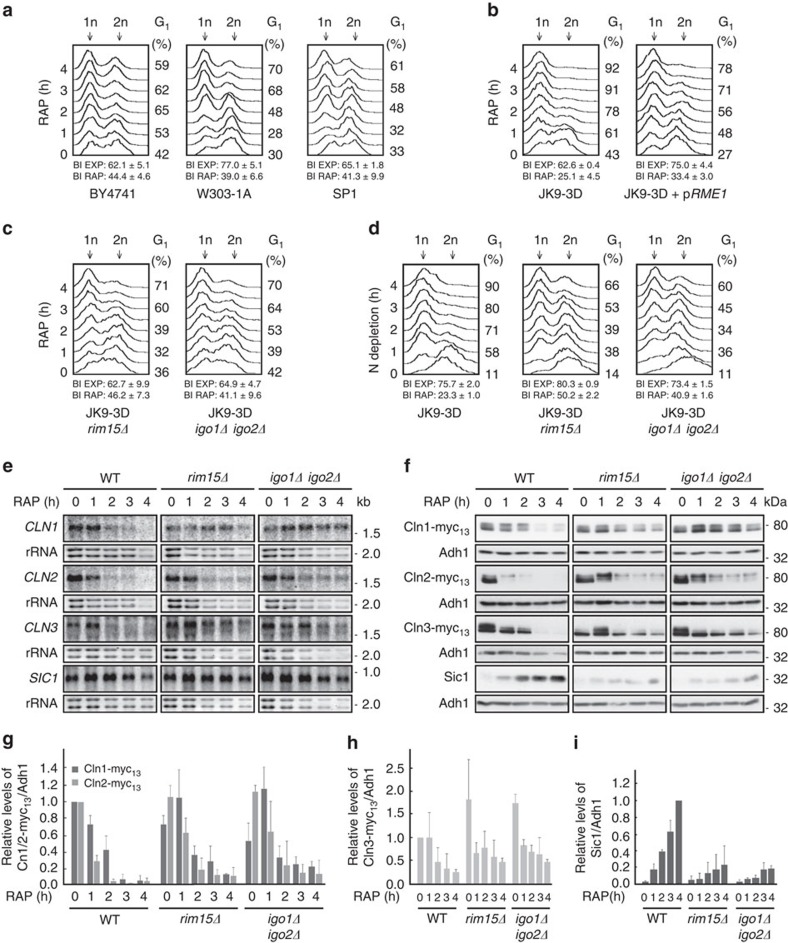
The greatwall kinase pathway ensures proper G_1_ arrest following TORC1 inactivation. (**a**) Cells of commonly used *Saccharomyces cerevisiae* wild-type strains (that is, BY4741, W303-1A and SP1) exhibit a delay in G_1_ arrest following rapamycin-mediated TORC1 inactivation. Fluorescence-activated cell sorting (FACS) analyses of the DNA content of wild-type cells treated for the indicated times with rapamycin are shown. The relative number of budded cells (budding index, BI) was determined in exponentially growing (EXP) and rapamycin-treated (RAP; 4 h) cultures. Numbers are means±s.d. from three independent experiments in which at least 300 cells were assessed. Populations of cells contain both 1n (G_1_; left-hand peak) and 2n (G_2_/M; right-hand peak) DNA. The relative level of 1n cells within the populations is indicated on the right of the graphs (G_1_ (%)). (**b**) JK9-3D cells promptly and uniformly arrest in G_1_ following rapamycin treatment. Expression of plasmid-encoded *RME1* from its own promoter (p*RME1*) delays the rapamycin-mediated G_1_ arrest in JK9-3D cells, indicating that the *rme1* mutation in JK9-3D contributes significantly to the observed phenotype. (**c**,**d**) Swift G_1_ arrest in rapamycin-treated (**c**) or nitrogen-starved (**d**) JK9-3D cells requires Rim15 and Igo1/2. (**e**) Northern blot analyses of the expression of the indicated cell cycle regulatory genes in exponentially growing (0 h) and rapamycin-treated (1–4 h) wild-type (WT; JK9-3D), *rim15*Δ and *igo1/2*Δ mutant cells. Ribosomal RNA served as loading control. (**f**–**i**) The levels of genomically myc_13_-tagged cyclins (**f**–**h**), or of endogenous Sic1 (**f**,**i**), in exponentially growing (0 h) and rapamycin-treated (1–4 h) WT (JK9-3D), *rim15*Δ and *igo1/2*Δ mutant cells, were determined by immunoblot analyses using monoclonal anti-myc or polyclonal anti-Sic1 antibodies, respectively. Adh1 levels served as loading controls. The experiments were performed independently three times (one representative blot is shown in **f**). The myc_13_-tagged cyclin (**g**,**h**) or Sic1 (**i**) levels were normalized to the Adh1 levels in each case, calculated relative to the value in exponentially growing WT cells (set to 1.0 (**g**,**h**)) or to the value in 4-h rapamycin-treated WT cells (set to 1.0 (**i**)), respectively, and expressed as mean values (*n*=3;±s.d.).

To study whether Rim15 mediates G_1_ cell cycle arrest via activation of endosulfines and consequently inhibition of PP2A^Cdc55^, we treated wild-type (WT) BY4741 cells with rapamycin and examined the cells by standard fluorescence-activated cell sorting (FACS) analyses. Unexpectedly, we found that BY4741 WT cells, like the ones from other commonly used WT strains such as W303-1A and SP1 (ref. 30), exhibited a significant delay in rapamycin-induced G_1_ arrest that contrasted with the quite rapid G_1_ arrest observed in JK9-3D WT cells (Fig. 1a,b). In trying to understand the different behaviour of JK9-3D cells, which have been instrumental for the discovery of TORC1 (ref. 31), we noticed that they carry a genomic *rme1* mutation that (on the basis of our complementation analysis) is in part responsible for their expedited rapamycin-induced G_1_ arrest (Fig. 1b). Of note, Rme1 contributes to G_1_ cyclin gene expression and has been assigned a specific role in preventing premature entry of cells into an off-cycle stationary phase (at G_1_) in response to nutrient limitation^32^. While this issue deserves to be addressed in more detail elsewhere, we decided to take advantage of the robust rapamycin-induced G_1_ arrest in JK9-3D cells to address our question whether Rim15 mediates G_1_ cell cycle arrest via activation of endosulfines. Accordingly, we found that a large fraction of *rim15*Δ and *igo1/2*Δ cells was significantly impaired in proper G_1_ arrest following rapamycin treatment when compared with their isogenic JK9-3D WT cells (Fig. 1c). This defect of *rim15*Δ and *igo1/2*Δ cells was even more pronounced following nitrogen starvation, a physiological condition that results in rapid TORC1 downregulation and subsequent G_1_ arrest in WT cells (Fig. 1d)^33^.

Since our results suggested a role for Rim15/Igo1/2 in cell cycle control, we next examined whether the expression of G_1_ cyclins (Cln1, Cln2 and Cln3) or of the CDKI Sic1 was altered in rapamycin-treated *rim15*Δ or *igo1/2*Δ mutant cells. In agreement with previous reports^6^,^7^, the *CLN1*–*3* transcripts and their corresponding proteins were progressively depleted in rapamycin-treated WT cells (Fig. 1e,f). In parallel, and consistent with the notion that TORC1 inhibition entails post-translational Sic1 stabilization^6^, Sic1 protein levels strongly increased despite the fact that the respective *SIC1* transcript levels remained relatively constant over the entire period of the rapamycin treatment. In rapamycin-treated *rim15*Δ and *igo1/2*Δ mutant cells, clearance of *CLN1–3* transcripts and of Cln1–3 proteins was noticeably delayed when compared with WT cells (Fig. 1e–h). In addition, loss of Rim15 or of Igo1/2, while only marginally affecting *SIC1* mRNA levels (Fig. 1e), severely and persistently compromised the ability of rapamycin-treated cells to accumulate Sic1 (Fig. 1f,i). This latter defect, which was also observed in respective BY4741, W303-1A and SP1 *rim15*Δ mutants (Supplementary Fig. 1), may in part be due to the delayed elimination of G_1_ cyclins that favour CDK-mediated multi-site phosphorylation and consequently SCF^Cdc4^-dependent ubiquitination and degradation of Sic1. However, both the transient nature of the G_1_ cyclin downregulation defect and the rather persistent Sic1 accumulation defect in rapamycin-treated *rim15*Δ and *igo1/2*Δ cells indicate that the Rim15-Igo1/2 signalling branch controls Sic1 stability also via an additional mechanism(s) that is not directly related to G_1_ cyclin expression control.

**Figure 2:**
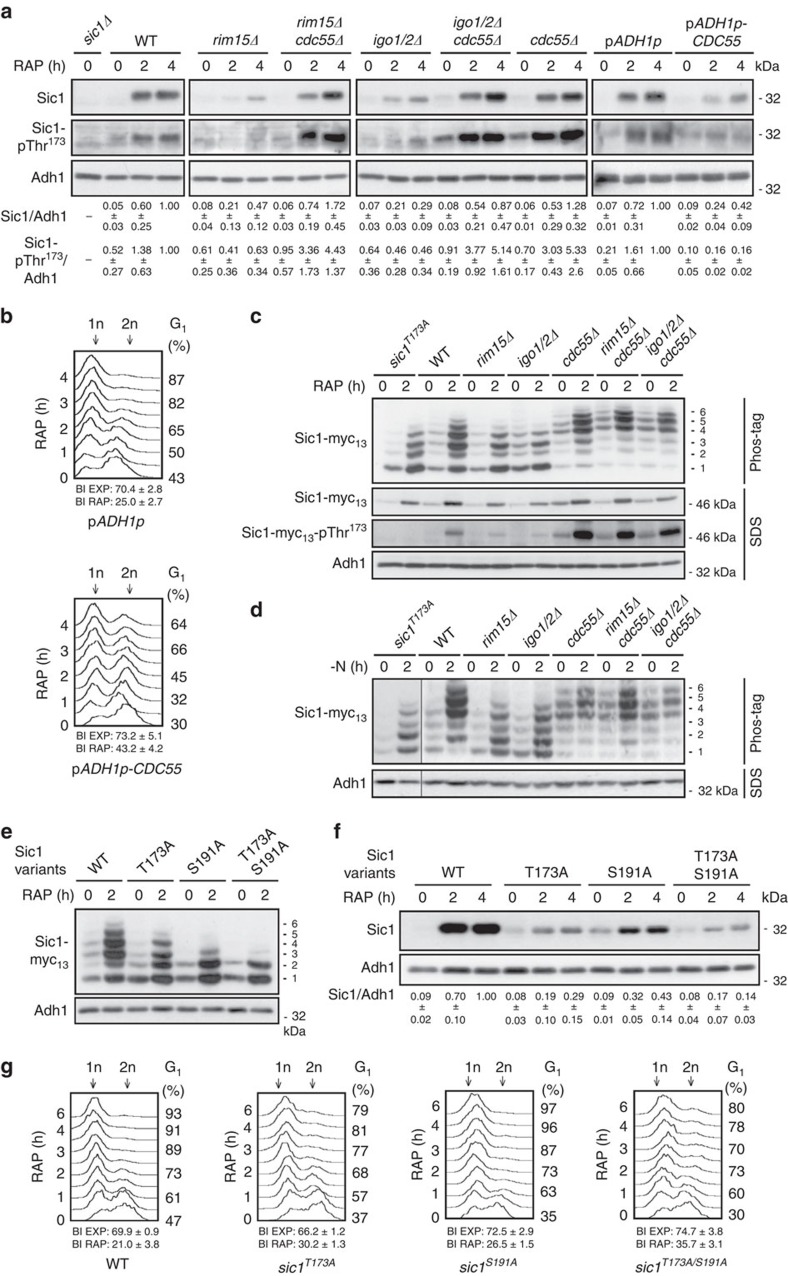
The greatwall kinase pathway regulates phosphorylation and stability of Sic1. (**a**) Loss of Cdc55 suppresses the defect of rapamycin-treated *rim15*Δ and *igo1/2*Δ cells in Sic1 accumulation. Sic1 levels and phosphorylation of Thr^173^ in Sic1 (Sic1-pThr^173^) were determined by immunoblot analyses using polyclonal anti-Sic1 and phospho-specific anti-Sic1-pThr^173^ antibodies, respectively. Overexpression of plasmid-encoded *CDC55* from the strong constitutive *ADH1* promoter (*ADH1p*) prevents normal Sic1 accumulation and reduces the total amount of Sic1-pThr^173^ in WT cells. Relevant genotypes are indicated. The experiments were performed independently three times (one representative blot is shown). The respective Sic1 levels or Sic1-pThr^173^ signals were normalized to the Adh1 levels in each case, calculated relative to the value in 4-h rapamycin-treated wild-type cells (set to 1.0), except for the values of the *CDC55*-overexpressing cells (p*ADH1p-CDC55*), which were calculated relative to the control cells carrying the empty vector (p*ADH1p*), and expressed as mean values (*n*=3;±s.d.). (**b**) Overexpression of plasmid-encoded *CDC55* from the *ADH1* promoter causes a substantial defect in G_1_ arrest in rapamycin-treated WT cells. (**c**–**e**) Phos-tag phosphate affinity gel electrophoresis analyses of genomically myc_13_-tagged Sic1, Sic1^T173A^, Sic1^S191A^ and/or Sic1^T173A/S191A^ in extracts from exponentially growing (time 0 h) and rapamycin-treated (RAP; 2 h; (**c**,**e**)) or nitrogen-deprived (−N; 2 h; (**d**)) strains with the indicated genotype. The six differentially phosphorylated Sic1-myc_13_ isoforms are numbered sequentially from 1 to 6 (right side of the panels). In **c**, samples were also subjected to SDS–gel electrophoresis to detect the Sic1-myc_13_ levels and Sic1-myc_13_-pThr^173^ signals by immunoblot analyses using monoclonal anti-myc and phospho-specific anti-Sic1-pThr^173^ antibodies, respectively. (**f**,**g**) The Sic1^T173A^ allele is unstable (**f**) and compromises timely G_1_ arrest in rapamycin-treated cells (**g**). Levels of Sic1 in **f** were determined in exponentially growing (time 0 h) and rapamycin-treated (2 and 4 h) WT, *sic1*^*T173A*^, *sic1*^*S191A*^ and *sic1*^*T173A/S191A*^ cells and quantified as in **a**. For quantifications of FACS profiles, see Supplementary Fig. 4. FACS and BI analyses in **b** and **g** were performed as in Fig. 1a. Adh1 levels in **a**,**c**,**d**,**e** and **f** served as loading controls. FACS, fluorescence-activated cell sorting.

Following their activation by Rim15, Igo1/2 mediate some, if not all, of their effects via the inhibition of PP2A^Cdc55^. Supporting this notion, we also found that loss of the regulatory Cdc55 subunit of the heterotrimeric PP2A^Cdc55^ complex rescued the Sic1 stabilization defect in rapamycin-treated *rim15*Δ and *igo1/2*Δ cells (Fig. 2a). Loss of Cdc55 alone, however, was not sufficient to drive Sic1 accumulation in exponentially growing cells, indicating that TORC1 antagonizes Sic1 by additional Cdc55-independent means. We were not able to examine whether loss of Cdc55 also suppresses the G_1_ arrest defect in rapamycin-treated *rim15*Δ or *igo1/2*Δ cells because all of the respective *cdc55*Δ mutants exhibited an extended G_2_/M delay that reflects an additional crucial role of PP2A^Cdc55^ in mitotic entry and spindle assembly checkpoint control^34^. Expectedly, however, overexpression of Cdc55 under the control of the constitutive *ADH1* promoter destabilized Sic1 (Fig. 2a) and caused a G_1_ arrest defect in rapamycin-treated cells (Fig. 2b) to a similar extent as loss of Rim15 or of Igo1/2 (Fig. 1c). Together with the current literature, these data could be unified in a model in which PP2A^Cdc55^ and Cln-CDK antagonize G_1_ arrest by favouring Sic1 destabilization via dephosphorylation and phosphorylation, respectively, of different, specific residues within Sic1.

### The greatwall kinase pathway impinges on Thr^173^ in Sic1

To begin to study how many residues in Sic1, if any, are targeted by PP2A^Cdc55^, we examined the migration pattern of Sic1-myc_13_ by phosphate affinity gel electrophoresis in different yeast strains. When analysed in extracts of exponentially growing WT, *rim15*Δ and *igo1/2*Δ cells, the weakly expressed Sic1-myc_13_ migrated in at least four distinct bands (labelled isoforms 1–4; Fig. 2c,d). Following rapamycin treatment (Fig. 2c), and similarly following nitrogen starvation (Fig. 2d), two additional slow-migrating Sic1-myc_13_ isoforms (labelled 5 and 6) were detectable in WT cell extracts. These were either absent (isoform 6) or reduced in intensity (isoform 5) in rapamycin-treated and in nitrogen-starved *rim15*Δ and *igo1/2*Δ cells, which likely explains the relative increase in the intensity of the faster migrating isoforms in the extracts of the respective strains (Fig. 2c,d). Loss of Cdc55, however, rendered *rim15*Δ and *igo1/2*Δ mutant cells capable again of expressing both isoforms (that is, isoforms 5 and 6) at levels comparably (or even higher) to the ones in WT cells under the same conditions. Of note, in exponentially growing, rapamycin-treated and nitrogen-starved cells, and independently of the presence or absence of Rim15 or Igo1/2, Sic1-myc_13_ preferentially migrated as isoforms 4, 5 and 6 when Cdc55 was absent. Together, these results indicate that PP2A^Cdc55^ targets at least two Sic1 phosphoresidues (to various degrees), and that activation of Rim15/Igo1/2 following rapamycin treatment or nitrogen starvation restrains the respective PP2A^Cdc55^ activity. To examine whether one of the respective residues corresponded to phosphorylated Thr^173^ (pThr^173^), which is critical for Sic1 stability in rapamycin-treated cells^6^ (Supplementary Fig. 2), we also analysed the migration pattern of a Sic1^T173A^ mutant allele via phosphate affinity gel electrophoresis in extracts of rapamycin-treated or nitrogen-starved cells. The Sic1^T173A^-myc_13_ migration pattern specifically lacked isoform 6 and was overall very similar to the one observed for Sic1-myc_13_ in extracts of rapamycin-treated or nitrogen-starved *rim15*Δ and *igo1/2*Δ mutant cells (Fig. 2c,d; Supplementary Fig. 3), indicating that pThr^173^ in Sic1 may indeed represent a PP2A^Cdc55^ target. Moreover, the previously identified phosphoresidue pSer^191^ in Sic1 (ref. 35) was required for the formation of three of the observed 6 isoforms (as Sic1^S191A^-myc_13_ migrated only in three (two major and one weaker) bands in rapamycin-treated cells; isoforms 1–3; Fig. 2e). Interestingly, mutation of Thr^173^ to Ala in Sic1 destabilized Sic1 and compromised proper G_1_ arrest in rapamycin-treated cells, and both of these defects were marginally enhanced by combined mutation of Thr^173^ and Ser^191^ to Ala in Sic1 (Fig. 2f,g; see budding indices; Supplementary Fig. 4). The Sic1^S191A^ allele *per se*, albeit less stable than WT Sic1, was able to ensure normal rapamycin-induced G_1_ arrest *in vivo* (Fig. 2f,g; Supplementary Fig. 4). The stability of Sic1 and hence proper G_1_ arrest in rapamycin-treated cells therefore primarily depend on the phosphorylation of Thr^173^ with at most accessory contributions from pSer^191^ (as well as potentially additional, less significant phosphoresidues). To further verify this assumption, we decided to focus our subsequent analyses on Thr^173^ in Sic1. Using phospho-specific antibodies against pThr^173^ in Sic1 (see below), we found that the Sic1-pThr^173^ signal strongly increased in rapamycin-treated WT cells, but not in Cdc55 overproducing nor in *rim15*Δ, or *igo1/2*Δ cells, unless the latter two mutant strains were additionally deleted for *CDC55* (Fig. 2a). In control experiments (corroborating the *in vivo* specificity of the anti-Sic1-pThr^173^ antibodies), mutation of Thr^173^ to Ala in Sic1 totally abolished the Sic1-pThr^173^ signal, and eliminated the Sic1-myc_13_ isoform 6 on phos-tag gels, even when Cdc55 was absent (Supplementary Fig. 3). Notably, Sic1-Thr^173^ phosphorylation signals closely mirrored the overall Sic1 levels in rapamycin-treated WT and all, except the *sic1*^*T173A*^, mutant strains tested (Fig. 2a). Together, these data corroborate a model in which activation of Rim15/Igo1/2 following TORC1 inhibition serves to antagonize PP2A^Cdc55^ and prevent it from dephosphorylating pThr^173^ (and possibly additional phosphoresidues) in Sic1, which presumably exposes Sic1 to a proteolytic degradation mechanism.

### Inactivation of SCF^Cdc4^ stabilizes Sic1^T173A^

**Figure 3:**
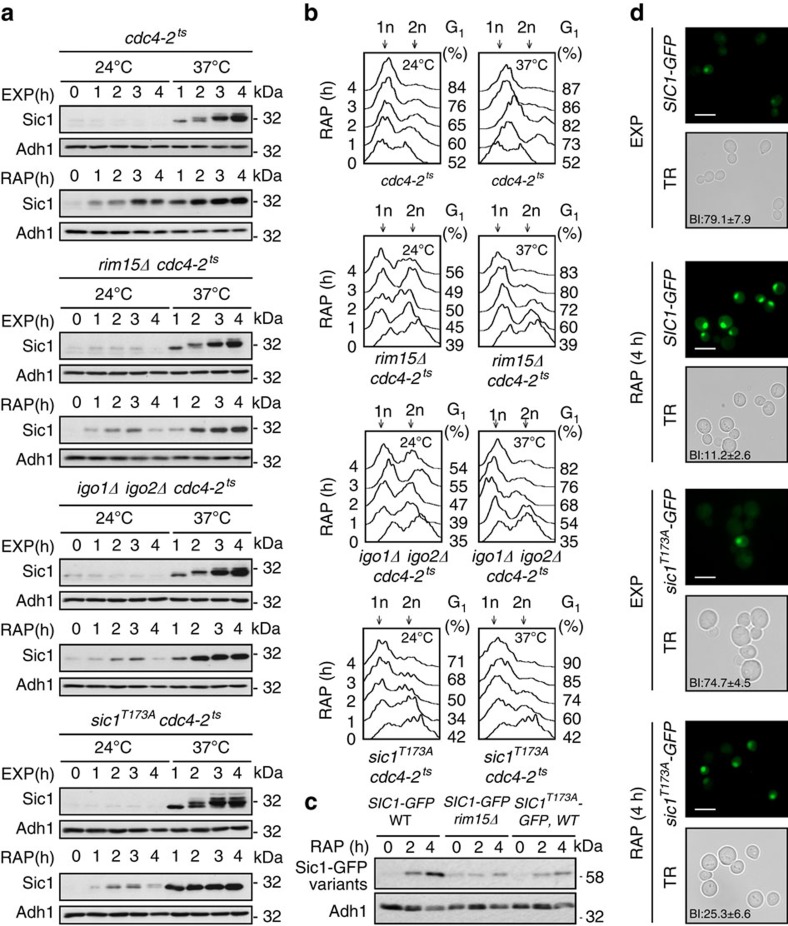
Inactivation of SCF^Cdc4^ stabilizes Sic1^T173A^. (**a**) Levels of endogenous Sic1 were determined by immunoblot analyses as in Fig. 2a. Cells (genotypes indicated) were pre-grown exponentially at 24 °C (time 0 h) and then grown up to 4 h at either 24 °C or 37 °C (to inactivate Cdc4-2^ts^) in the absence (EXP) or presence of rapamycin (RAP). Samples were taken at the indicated time points. (**b**) FACS analyses from cells treated as in **a**. (**c**,**d**) Sic1-Thr^173^ phosphorylation primarily serves to control Sic1 stability, but not Sic1 subcellular localization. In **c**, levels of endogenously tagged Sic1–GFP and Sic1^T173A^–GFP were determined in exponentially growing (time 0 h) and rapamycin-treated (2 h and 4 h) WT and/or *rim15*Δ cells by immunoblot analyses using polyclonal anti-GFP antibodies. In **d**, exponentially growing (EXP) or rapamycin-treated (RAP; 4 h) cells expressing endogenously tagged versions of Sic1–GFP or Sic1^T173A^–GFP were analysed by fluorescence microscopy. Scale bars, 5 μm (white); TR, transmission; BI, budding index. Adh1 levels in **a** and **c** served as loading controls. FACS, fluorescence-activated cell sorting.

To examine whether phosphorylation of Thr^173^ in Sic1 may serve to protect Sic1 from SCF^Cdc4^-mediated ubiquitination and subsequent proteolysis, we introduced the temperature-sensitive *cdc4-2*^*ts*^ allele in our WT, *rim15*Δ, *igo1/2*Δ and *sic1*^*T173A*^ strains, and measured their capacity to accumulate Sic1 during exponential growth or following rapamycin treatment at the permissive (24 °C) and the non-permissive temperature (37 °C). At 24 °C, the *cdc4-2*^*ts*^ allele did not noticeably alter the Sic1 expression pattern in any of the strains studied, whether they were grown exponentially or subjected to rapamycin treatment (that is, specifically *rim15*Δ *cdc4-2*^*ts*^, *igo1/2*Δ *cdc4-2*^*ts*^ and *sic1*^*T173A*^ *cdc4-2*^*ts*^ cells were still defective for normal Sic1 accumulation following rapamycin treatment when compared with *cdc4-2*^*ts*^ cells; Fig. 3a). Temperature inactivation of Cdc4-2^ts^ (at 37 °C), however, prompted Sic1 accumulation to a similarly strong extent in all strains, independently of the presence or absence of rapamycin, and could thus override the defect in Sic1 accumulation, but not in Sic1-Thr^173^ phosphorylation (Supplementary Fig. 5a,b), in rapamycin-treated *rim15*Δ *cdc4-2*^*ts*^, *igo1/2*Δ *cdc4-2*^*ts*^, and *sic1*^*T173A*^ *cdc4-2*^*ts*^ cells. As expected, the latter mutant strains also regained their capacity to timely arrest in G_1_ following rapamycin treatment at 37 °C, but not at 24 °C (Fig. 3b). Rim15-Igo1/2-mediated inhibition of PP2A^Cdc55^ therefore likely serves to preserve the phosphorylation status of Thr^173^ (and other residues) in Sic1, thereby preventing SCF^Cdc4^-mediated ubiquitination and subsequent proteolysis of Sic1. To address the possibility that Sic1-Thr^173^ phosphorylation plays an additional role in nutrient-regulated nucleo-cytoplasmic distribution of Sic1 (ref. 36), we examined the localization of endogenously tagged Sic1–green fluorescent protein (GFP) and of Sic1^T173A^–GFP. These GFP fusions behaved like the respective untagged versions in terms of their stability (that is, Sic1–GFP accumulated in rapamycin-treated WT, but not in *rim15*Δ cells, and Sic1^T173A^–GFP was intrinsically unstable in a WT context under the same conditions; Fig. 3c). Sic1^T173A^–GFP, although expressed at lower levels and compromised in ensuring proper G_1_ arrest to a larger fraction of the population, was able to accumulate like Sic1–GFP within the nuclei of those cells that were still able to arrest in an unbudded state, specifically also following rapamycin treatment (Fig. 3d). Thus, Sic1-Thr^173^ phosphorylation likely serves to primarily control Sic1 stability, but not Sic1 subcellular localization.

### Mpk1 phosphorylates Thr^173^ in Sic1

**Figure 4:**
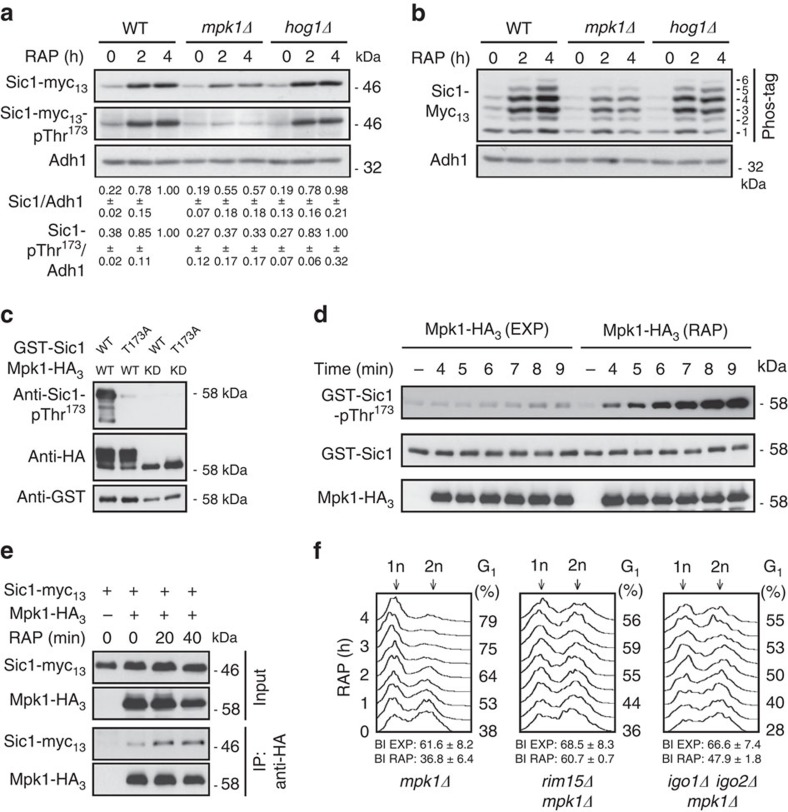
Mpk1 phosphorylates Thr^173^ in Sic1. (**a**) Mpk1, but not Hog1, is required for normal Sic1 accumulation in rapamycin-treated cells. Levels of Sic1-myc_13_ and of Sic1-myc_13_-pThr^173^ signals were determined in cells with the indicated genotypes before (0 h) and following a rapamycin treatment (for 2 and 4 h). The Sic1-myc_13_ levels or Sic1-myc_13_-pThr^173^ signals (three independent experiments) were normalized to Adh1 in each case, calculated relative to the value in 4-h rapamycin-treated wild-type cells (set to 1.0), and expressed as mean values (±s.d.). (**b**) Phos-tag phosphate affinity gel electrophoresis analysis of genomically myc_13_-tagged Sic1 from exponentially growing (time 0 h) and rapamycin-treated (2 and 4 h) WT, *mpk1*Δ and *hog1*Δ cells were carried out as in Fig. 2c. (**c**) Mpk1 phosphorylates Thr^173^ in Sic1 *in vitro*. Mpk1-HA_3_ and kinase-dead Mpk1^KD^-HA_3_ (carrying the K54R mutation) were purified from rapamycin-treated (1 h) cells and used for *in vitro* protein kinase assays on bacterially purified GST-Sic1 or GST-Sic1^T173A^. Levels of Sic1 protein and of Sic1-pThr^173^ signals were determined using anti-GST and anti-Sic1-pThr^173^ antibodies, respectively. Immunoblot analysis using anti-HA antibodies served as input control for Mpk1-HA_3_ variants. Mpk1-HA_3_, but not Mpk1^KD^-HA_3_, displayed slow-migrating isoforms due to post-translational modifications. (**d**) Rapamycin treatment strongly stimulates Mpk1 protein kinase activity towards Thr^173^ in Sic1. *In vitro* protein kinase assays were carried out as in **c** for the indicated times using Mpk1-HA_3_ preparations from exponentially growing (EXP) or rapamycin-treated (1 h; RAP) cells. (**e**) Rapamycin treatment stimulates the interaction between Sic1-myc_13_ and Mpk1-HA_3_. Plasmid-encoded Mpk1-HA_3_ was immunoprecipitated from extracts of untreated (0 min) and rapamycin-treated (RAP; 20 and 40 min) Sic1-myc_13_-expressing WT cells. Cells carrying an empty vector (−) were used as control. The co-precipitated Sic1-myc_13_ levels were detected by immunoblot analysis using anti-myc antibodies. (**f**) Proper G_1_ arrest in rapamycin-treated cells requires Mpk1. FACS analyses (see Supplementary Fig. 6 for quantifications of triplicates) and BI determinations were performed as in Fig. 1a. All strains (relevant genotypes indicated) are isogenic to JK9-3D (see Fig. 1b,c for comparison). FACS, fluorescence-activated cell sorting.

Since rapamycin treatment was able to strongly increase the Sic1-pThr^173^ signal in *cdc55*Δ cells (Fig. 2a), we reasoned that TORC1 is additionally involved in downregulation of a Sic1-Thr^173^-targeting protein kinase(s). In this context, the MAPK Hog1 has previously been proposed to mediate Sic1-Thr^173^ phosphorylation following exposure of cells to osmotic stress^37^. Whether TORC1 impinges on Hog1 is not known, but TORC1 indirectly inhibits the closely related MAPK Slt2/Mpk1 (refs 38, 39). Intriguingly, and consistent with a role of Mpk1 in Sic1-Thr^173^ phosphorylation, loss of Mpk1, but not of Hog1, significantly reduced the Sic1-pThr^173^ signal and rendered Sic1 unstable in rapamycin-treated cells (Fig. 4a). Moreover, the migration pattern of Sic1-myc_13_ (analysed by phosphate affinity gel electrophoresis) in extracts of rapamycin-treated WT, *mpk1*Δ and *hog1*Δ cells indicated that Mpk1, but not Hog1, might (directly or indirectly) target one major residue in Sic1 (compare the levels of isoforms 5 and 6 in WT and *hog1*Δ versus *mpk1*Δ cells in Fig. 4b). Together, these data pinpoint a potential role for Mpk1 in direct phosphorylation of Thr^173^ in Sic1. Corroborating this assumption, we further found that Mpk1-HA_3_, but not kinase-dead Mpk1^KD^-HA_3_, strongly phosphorylated Sic1-Thr^173^ *in vitro* (Fig. 4c; notably, the respective signal was almost entirely abrogated by introduction of the Thr^173^ to Ala mutation in Sic1, indicating that the anti-Sic1-pThr^173^ antibodies are also exquisitely specific *in vitro*). In addition, rapamycin treatment not only stimulated the activity of Mpk1 towards Thr^173^ in Sic1 10.4-fold (±2.6 s.d.; three independent time-course experiments; Fig. 4d), but also significantly boosted the interaction of Mpk1 with Sic1 *in vivo* (Fig. 4e). From these studies, we infer that Mpk1 directly phosphorylates Sic1-Thr^173^ *in vivo*, thereby contributing to Sic1 stability when TORC1 is attenuated. Expectedly, therefore, loss of Mpk1, but not of Hog1, also caused a significant G_1_ arrest defect in rapamycin-treated cells (Fig. 4f; Supplementary Fig. 6).

### Mpk1 and PP2A^Cdc55^ reciprocally control Sic1-pThr^173^

**Figure 5:**
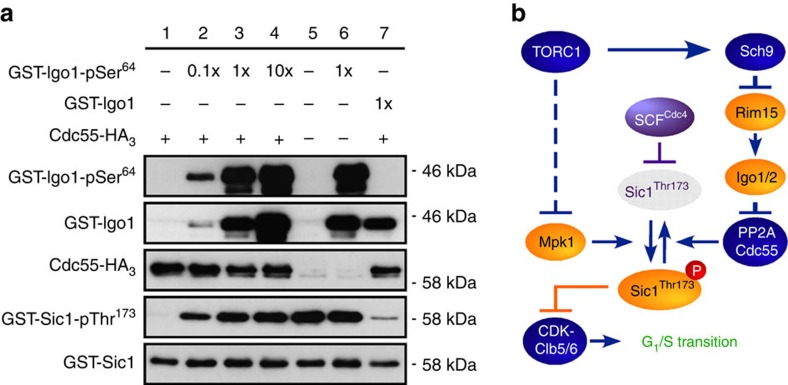
TORC1 coordinates G_1_–S cell cycle progression via Mpk1 and PP2A^Cdc55^. (**a**) PP2A^Cdc55^ dephosphorylates pThr^173^ in Sic1 and this activity is inhibited in a concentration-dependent manner by activated Igo1 (Igo1-pSer^64^), but not inactive Igo1. GST-Sic1 was phosphorylated by Mpk1 *in vitro* before being used as a substrate for the PP2A^Cdc55^ phosphatase assay. Phosphatase activity of PP2A^Cdc55^ was analysed in the absence (lane 1) and in the presence of increasing amounts (lanes 2, 3 and 4, respectively) of recombinant Igo1-pSer^64^, which had been subjected to thio-phosphorylation by Rim15 previously. Assays without both PP2A^Cdc55^ and Igo1-pSer^64^ (lane 5), without PP2A^Cdc55^ but with Igo1-pSer^64^ (lane 6), and with PP2A^Cdc55^ combined with inactive Igo1 (lane 7) were included as additional controls. The levels of Ser^64^ phosphorylation in GST-Igo1 (GST-Igo1-pSer^64^), GST-Igo1, Cdc55-HA_3_, Thr^173^ phosphorylation in GST-Sic1 (GST-Sic1-pThr^173^) and GST-Sic1 were determined by immunoblot analyses using phospho-specific anti-Igo1-pSer^64^, anti-GST, anti-HA, phospho-specific anti-Sic1-pThr^173^ and anti-GST antibodies, respectively. (**b**) Model for the role of TORC1 in regulating the phosphorylation status and stability of the CDKI Sic1. For the sake of clarity, we have not schematically depicted the additional role of Rim15-Igo1/2 in G_1_ cyclin downregulation that may transiently favour CDK-mediated multi-site phosphorylation and consequently SCF^Cdc4^-dependent ubiquitination and degradation of Sic1 following TORC1 inactivation. Sic1 inhibits the CDK–Clb5/6 complexes to prevent transition into S phase^43^. Arrows and bars denote positive and negative interactions, respectively. Solid arrows and bars refer to direct interactions, the dashed bar refers to an indirect interaction. For details see text.

Since we were able to phosphorylate Sic1-Thr^173^ with Mpk1, we also examined whether PP2A^Cdc55^ could directly dephosphorylate this residue *in vitro*. As illustrated in Fig. 5a, PP2A^Cdc55^ indeed very efficiently dephosphorylated pThr^173^ in Sic1 in these assays (Fig. 5a, lane 1 versus lane 5). In addition, following prior activation by Rim15, Igo1 (Igo1-pSer^64^; Fig. 5a, lanes 2–4), but not inactive Igo1 (Fig. 5a, lane 7), efficiently inhibited the respective PP2A^Cdc55^ activity in a concentration-dependent manner. Thus, Mpk1 and PP2A^Cdc55^ directly and antagonistically control the phosphorylation status of Thr^173^ in Sic1 both *in vitro* and within cells. Of note, since Sic1-Thr^173^ phosphorylation was not fully abolished in the absence of Mpk1 (in *mpk1*Δ), nor in the presence of unrestricted PP2A^Cdc55^ (in *rim15*Δ or *igo1/2*Δ cells), we expected the combination of *mpk1*Δ with either *rim15*Δ or *igo1/2*Δ to cause an additive G_1_ arrest defect in rapamycin-treated cells. This was indeed the case (Fig. 4f).

## Discussion

TORC1 coordinates START with nutrient availability in part by tightly regulating the phosphorylation status of Thr^173^ within the CDKI Sic1 (Fig. 5b). Together with the previous observations (i) that Sic1 only marginally interacts with the catalytic SCF^Cdc4^ subunit Cdc34 in rapamycin-treated cells^6^ and (ii) that the introduction of a phosphomimetic Glu at position 173 of Sic1 compromises its capacity to interact with Cdc4 (ref. 37), our present data are best explained in a model in which phosphorylation of Thr^173^ in Sic1 serves to stabilize Sic1 by preventing (directly or indirectly) its association with SCF^Cdc4^. Of note, Cln-CDK downregulation following TORC1 inhibition, which transiently relies on Rim15 and Igo1/2 (Fig. 1e,f), presumably also contributes to the latter process. It will therefore be interesting in future studies to decipher the respective Rim15- and Igo1/2-dependent and -independent mechanism(s) by which TORC1 controls transcriptional and/or post-transcriptional control of G_1_ cyclin expression.

Finally, Sic1 is functionally and structurally related to the mammalian CDKI p27^Kip1^, an atypical tumour suppressor that regulates the G_0_–S cell cycle transition by inhibiting cyclin-CDK2-containing complexes^40^. Similar to Sic1, p27^Kip1^ turnover is stimulated by direct cyclin-CDK2-mediated phosphorylation, followed by SCF^Skp2^-dependent ubiquitination and proteasomal degradation in proliferating cells. In quiescent G_0_ cells, in contrast, phosphorylation of specific alternative residues ensures p27^Kip1^ stability^40^. Since p27^Kip1^ also mediates in part the anti-proliferative effects of rapamycin^4^, it will be interesting to study whether and to what extent our findings in yeast may have been evolutionarily conserved.

## Methods

### Strains, plasmids and growth conditions

*Saccharomyces cerevisiae* yeast cells were pre-grown overnight at 30 °C in standard synthetic defined (SD) medium with 2% glucose and supplemented with the appropriate amino acids for maintenance of plasmids. Before the experiments, cells were diluted to an OD_600_ of 0.001 in SD and grown until they reached an OD_600_ of 0.4. Rapamycin was dissolved in 10% Tween-20/90% ethanol and used at a final concentration of 200 ng ml^−1^. Strains and plasmids used in this study are listed in Supplementary Tables 1 and 2, respectively. Epitope-tagged proteins studied were expressed from their genomic locus, except GST-Sic1, Mpk1-HA_3_ and Cdc55-HA_3_ that were expressed from plasmids (under the control of their own promoter) to be used for the *in vitro* protein kinase and phosphatase assays.

### Fluorescence-activated cell sorting analysis

A measure of 1.5 ml samples were collected at the indicated time points after rapamycin treatment, centrifuged and resuspended in 1 ml 70% ethanol. Following overnight incubation at 4 °C, cells were washed once with H_2_O, centrifuged, resuspended in 250 μl of RNAse solution (50 mM Tris (pH 7.4), 200 μg ml^−1^ RNAse A (Axonlab AG)) and incubated for 3 h at 37 °C. Subsequently, cells were centrifuged again, resuspended in 250 μl of propidium idodide solution (50 mM Na^+^-citrate (pH 7.0) and 10 μg ml^−1^ propidium idodide (Sigma)) and analysed in a CyFlow (PARTEC) flow cytometer. Data were processed using the FlowJo software.

### Northern blot and immunoblot analyses

Northern blot analyses were performed according to our standard protocol^18^ and the respective uncropped scans have been included in Supplementary Fig. 7. Total protein extracts were prepared by mild alkali treatment of cells followed by boiling in standard electrophoresis buffer^41^. SDS–polyacrylamide gel electrophoresis and immunoblot analyses were performed according to standard protocols. For the analysis of protein phosphorylation states, we used Phos-tag acrylamide gel electrophoresis^42^. Anti-Sic1, (sc-50441; Santa Cruz), anti-c-Myc (9E10; sc-40; Santa Cruz), anti-Adh1 (Calbiochem), phospho-specific anti-Sic1-pThr^173^ (produced by GenScript), anti-GFP (Roche), phospho-specific anti-Igo1-pSer^64^ (ref. 24), anti-GST (Lubio) and anti-HA antibodies (Enzo) were used at 1:1,000, 1:3,000, 1:200,000, 1:1,000, 1:3,000, 1:1,000, 1:1,000 and 1:1,000 dilutions, respectively. Goat anti-rabbit/anti-mouse IgG-horseradish peroxidase-conjugated antibodies (BioRad) were used at a 1:3,000 dilution. All immunoblots presented in the main text have been included as uncropped scans in Supplementary Figs 8–25.

### Co-immunoprecipitation

For co-immunoprecipitation analyses, Sic1-myc_13_- and Mpk1-HA_3_-expressing cells were fixed for 20 min with 1% formaldehyde, quenched with 0.3 M glycine, washed once with Tris-buffered saline, centrifuged and subsequently frozen (−80 °C). Lysates were prepared by disruption of frozen cells in lysis buffer (50 mM TRIS (pH 7.5), 1 mM EDTA, 150 mM NaCl, 0.5% NP40 and 1 × protease and phosphatase inhibitor cocktails (Roche)) with glass beads (0.5-mm diameter) using a Precellys cell disruptor and subsequent clarification by centrifugation (5 min at 14,000 r.p.m.; 4 °C). Mpk1-HA_3_ was immunoprecipated with anti-HA magnetic matrix (Pierce) and co-immunoprecipitated Sic1-myc_13_ was determined by immunoblot analysis using anti-c-Myc antibodies.

### Mpk1 protein kinase assays

Mpk1-HA_3_ or Mpk1^K54R^ -HA_3_was immunopurified from yeast cells using anti-HA magnetic matrix (Pierce). The respective matrices were incubated for 30 min at 30 °C with 3 μl of bacterially purified GST-Sic1 or GST-Sic1^T173A^ in 50 μl of kinase buffer mix (125 mM Tris (pH 7.5), 50 mM MgCl_2_, 2.5 mM dithiothreitol and 10 mM ATP). The reactions were stopped by addition of loading buffer, boiled at 95 °C and analysed by immunoblot analyses. For the Mpk1 kinase time-course experiment, Mpk1-HA_3_ was purified from exponentially growing or rapamycin-treated (1 h) cells. The protein kinase reactions (with bacterially purified GST-Sic1 as substrate) were stopped at the indicated time points by addition of loading buffer and subsequent boiling (5 min).

### PP2A^Cdc55^ protein phosphatase assay

Cdc55-HA_3_ was isolated from exponentially growing *cdc55*Δ cells carrying the pRS416-*CDC55-HA*_*3*_ plasmid. Cdc55-HA_3_ was immunoprecipitated from total extracts in lysis buffer (50 mM Tris (pH 7.5), 1 mM EDTA, 150 mM NaCl, 0.5% NP40 and 1 × protease and phosphatase inhibitor cocktails from Roche) using anti-HA magnetic matrix (Pierce). Igo1-GST and Igo1^S64A^-GST were isolated from bacteria using glutathione sepharose (GE Healthcare) and phosphorylated where indicated by yeast-purified GST-Rim15-HA_3_ using 1 mM adenosine 5'-[γ-thio] triphosphate^17^,^22^. The *in vitro* phosphatase assay (30 min at 30 °C) was performed in phosphatase buffer (10 mM Tris (pH 7.5), 5 mM MgCl_2_ and 1 mM EGTA) with purified PP2A^Cdc55^, bacterially purified Sic1-GST that was phosphorylated by Mpk1 *in vitro* as substrate, and different concentrations of Igo1, which was, or was not, subjected to *in vitro* phosphorylation by Rim15 before the use. To assess PP2A^Cdc55^ activity, the decrease in Sic1^T173^ phosphorylation was detected using phospho-specific anti-Sic1-pThr^173^ antibodies. Levels of immunoprecipitated Cdc55-HA_3_ were assessed using anti-HA antibodies.

## Additional information

**How to cite this article:** Moreno-Torres, M. *et al*. TORC1 controls G1–S cell cycle transition in yeast via Mpk1 and the greatwall kinase pathway. *Nat. Commun.* 6:8256 doi: 10.1038/ncomms9256 (2015).

## Supplementary Material

Supplementary Information Supplementary Figures 1-25, Supplementary Tables 1-2 and Supplementary References
