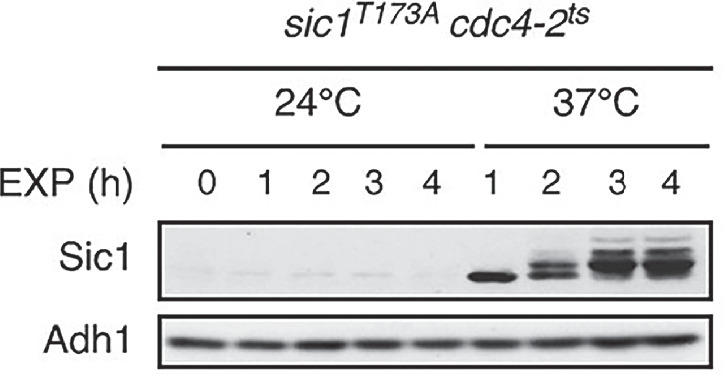# Author Correction: TORC1 controls G_1_–S cell cycle transition in yeast via Mpk1 and the greatwall kinase pathway

**DOI:** 10.1038/s41467-026-71875-1

**Published:** 2026-05-11

**Authors:** Marta Moreno-Torres, Malika Jaquenoud, Claudio De Virgilio

**Affiliations:** Department of Biology, University of Fribourg, Chemin du Muse´e 10, Fribourg, CH-1700 Switzerland

Correction to: *Nature Communications* 10.1038/ncomms9256, published online 10 September 2015

In the version of the article initially published, in Fig. 3a, the “Adh1” image for “*sic1*^*T173A*^ *cdc4-2*^*ts*^” (EXP) was a duplicate of the “Adh1”, “*igo1∆ igo2∆ cdc4-2*^*ts*^” (RAP) image. However, the correct, original data (loading control) was included in the Supplementary Information published alongside the original article (Supplementary Fig. 14). The correct image is included below as Fig. 1. This notice serves to amend the error.

**Fig. 1** Corrected Fig. 3a (*sic1*^*T173A*^ *cdc4-2*^*ts*^ EXP panel)