# Supplementary material for: TORC1 controls G1–S cell cycle transition in yeast via Mpk1 and the greatwall kinase pathway
Source: Nat Commun. 2015 Sep 10;6:8256. doi: 10.1038/ncomms9256 (PMC4579850; doi:10.1038/ncomms9256)
Supplement: Supplementary Information — Supplementary Figures 1-25, Supplementary Tables 1-2 and Supplementary References [file ncomms9256-s1.pdf]

Supplementary Figures

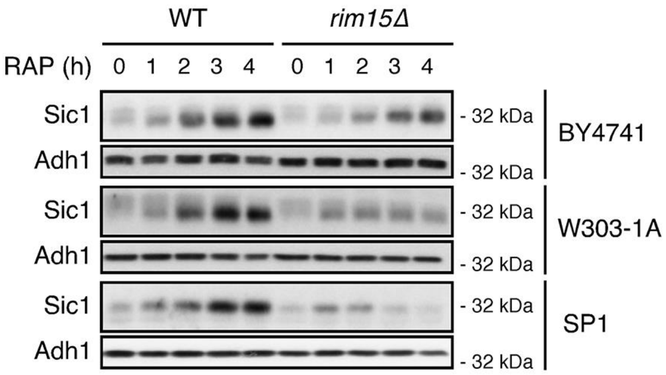

Supplementary Figure 1 | Rim15 ensures Sic1 accumulation following TORC1 inactivation independently of the yeast strain background. The levels of endogenous Sic1, in exponentially growing (0 h) and rapamycin-treated (RAP; 1-4 h) BY4741, W303-1A, and SP1 wild-type (WT) and respective isogenic *rim15*Δ mutant cells, were determined by immunoblot analyses using polyclonal anti-Sic1 antibodies. Adh1 levels served as loading controls.

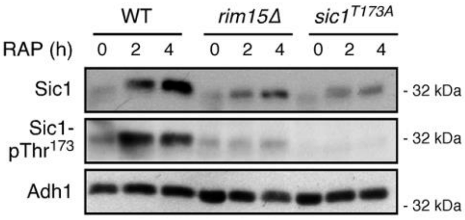

Supplementary Figure 2 | Mutation of Thr[173] to Ala in Sic1, like loss of Rim15, compromises normal Sic1 accumulation in rapamycin-treated cells. Sic1 levels and phosphorylation of Thr[173] in Sic1 (Sic1-pThr[173]) were determined in exponentially growing (0 h) and rapamycin-treated (RAP; 2 and 4 h) cells with the indicated genotypes by immunoblot analyses using polyclonal anti-Sic1 and phosphospecific anti-Sic1-pThr[173] antibodies, respectively. Adh1 levels served as loading controls.

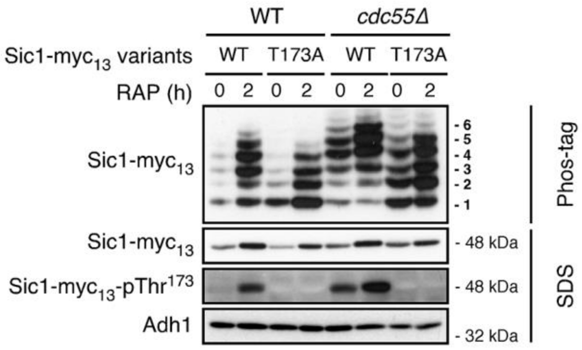

Supplementary Figure 3 | Phos-tag phosphate affinity gel electrophoresis analysis of genomically myc$_{13}$-tagged Sic1 or Sic1^{T173A}. Sic1-myc$_{13}$ or Sic1^{T173A}-myc$_{13}$ were analyzed by phos-tag phosphate affinity and SDS gel electrophoresis (followed by immunoblot analysis using anti-myc or anti-Sic1-pThr[173] antibodies) in extracts from exponentially growing (0 h) and rapamycin-treated (RAP; 2 h) WT and *cdc55*Δ strains. The 6 differentially phosphorylated Sic1-myc$_{13}$ isoforms are numbered sequentially from 1 to 6 (right side of the panels). Adh1 levels served as loading controls.

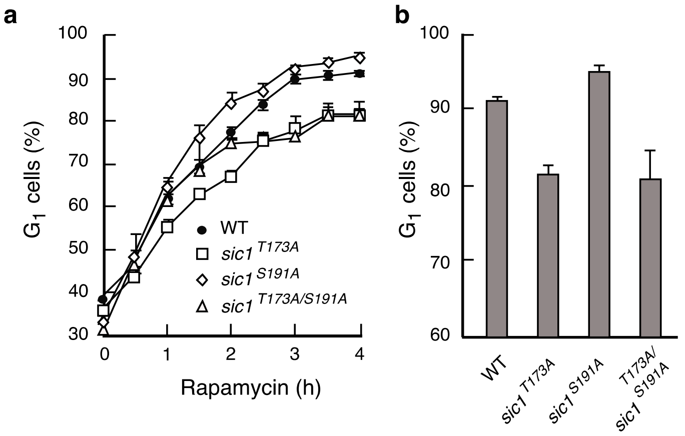

Supplementary Figure 4 | The Sic1^{T173A} allele compromises G$_1$ arrest in rapamycin-treated cells. (**a**) FACS analyses were performed in exponentially growing (0 h) and rapamycin-treated (times indicated) WT, *sic1^{T173A}*, *sic1^{S191A}*, and *sic1$^{T173A/S191A}$* cells. The experiments were performed independently 3 times for each strain (one representative FACS profile is shown in Fig. 2g) and the quantifications (means ± SD) of the percentage of G$_1$ cells in the respective populations are presented. (**b**) Bar graphs show the percentage of G$_1$ cells in the populations of rapamycin-treated (4 h) strains with the indicated genotypes with error bars indicating the 95% confidence interval. The data points from the 4-h rapamycin treatment were further used to perform an ANOVA analysis, which was followed by a Tukey's post-hoc test to examine the differences for each pair of strains. We found a highly significant difference among the four strains (ANOVA, p-value <0.001). Tukey's post-hoc test indicated that the values for WT and *sic1^{S191A}* cells were not significantly different from each other (p-value=0.133); similarly the values for *sic1^{T173A}* and *sic1$^{T173A/S191A}$*cells were also not significantly different from each other (p-value=0.97). All other pairwise comparisons were statistically significant (all p-values <0.001), showing that the values for WT and *sic1^{S191A}* cells significantly diverged from the ones of the *sic1^{T173A}* and *sic1$^{T173A/S191A}$* cells.

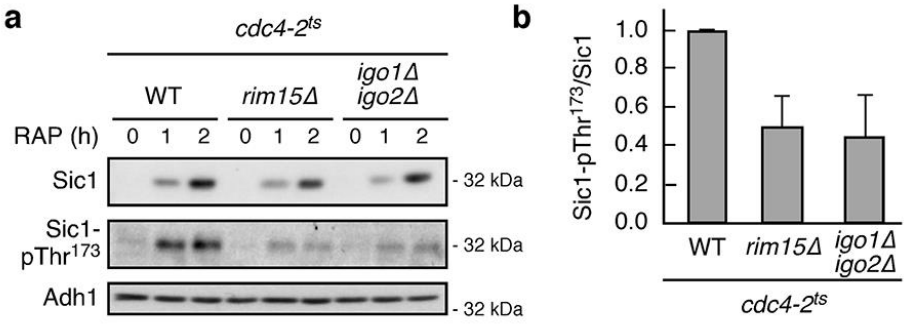

Supplementary Figure 5 | Inactivation of the SCF^{Cdc4} ubiquitin ligase suppresses the defect in Sic1 accumulation, but not in Sic1-Thr^{173} phosphorylation, in rapamycin-treated *rim15*Δ *cdc4-2^{ts}* and *igo1*Δ *igo2*Δ *cdc4-2^{ts}*mutant cells. (**a**) Sic1 levels and phosphorylation of Thr^{173} in Sic1 (Sic1-pThr^{173}) were determined by immunoblot analyses using polyclonal anti-Sic1 and phosphospecific anti-Sic1-pThr^{173} antibodies, respectively. Cells (genotypes indicated) were pre-grown exponentially at 24°C (0 h) and then shifted to 37°C for 1 or 2 h (to inactivate Cdc4-2^{ts}) in the presence of rapamycin (RAP). Adh1 levels served as loading controls. The experiment was performed independently 3 times and one representative set of blots is shown. (**b**) Bars represent the ratio between the mean Sic1-pThr^{173} levels and Sic1 protein levels (± SD; 3 independent experiments), determined in rapamycin-treated (2h at 37°C) *cdc4-2^{ts}*, *rim15*Δ *cdc4-2^{ts}*, and *igo1*Δ *igo2*Δ *cdc4-2^{ts}* cells and expressed relative to the value in *cdc4-2^{ts}* cells (set to 1.0).

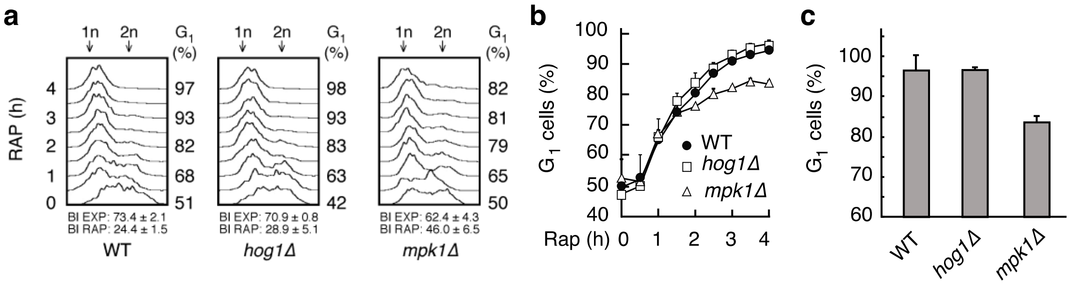

Supplementary Figure 6 | Loss of Mpk1, but not of Hog1, compromises timely G_1 arrest in rapamycin-treated cells. (**a**) FACS analyses were performed in exponentially growing (time 0 h) and rapamycin-treated (times indicated) WT, *hog1*Δ, and *mpk1*Δ cells. FACS and BI analyses were performed as in Fig. 1a. The experiments were performed independently 3 times for each strain (one representative FACS profile is shown). (**b**) Quantifications (means ± SD) of the percentage of G_1 cells in the respective populations in (a) are presented. (**c**) Bar graphs showing the percentage of G_1 cells in the populations of rapamycin-treated (4 h) strains with the indicated genotypes with error bars indicating the 95% confidence interval. The data points from the 4-h rapamycin treatment were further used to perform an ANOVA analysis, which was followed by a Tukey's post-hoc test to examine the differences for each pair of strains. We found a highly significant difference among the three strains (ANOVA, p-value <0.001). Tukey's post-hoc test indicated that the values for WT and *hog1*Δ cells were not significantly different from each other (p-value=0.53), but that the values for the *mpk1*Δ cells were significantly different from the other two strains (both p-values <0.002).

Supplementary Figures 7-25: Original Blots

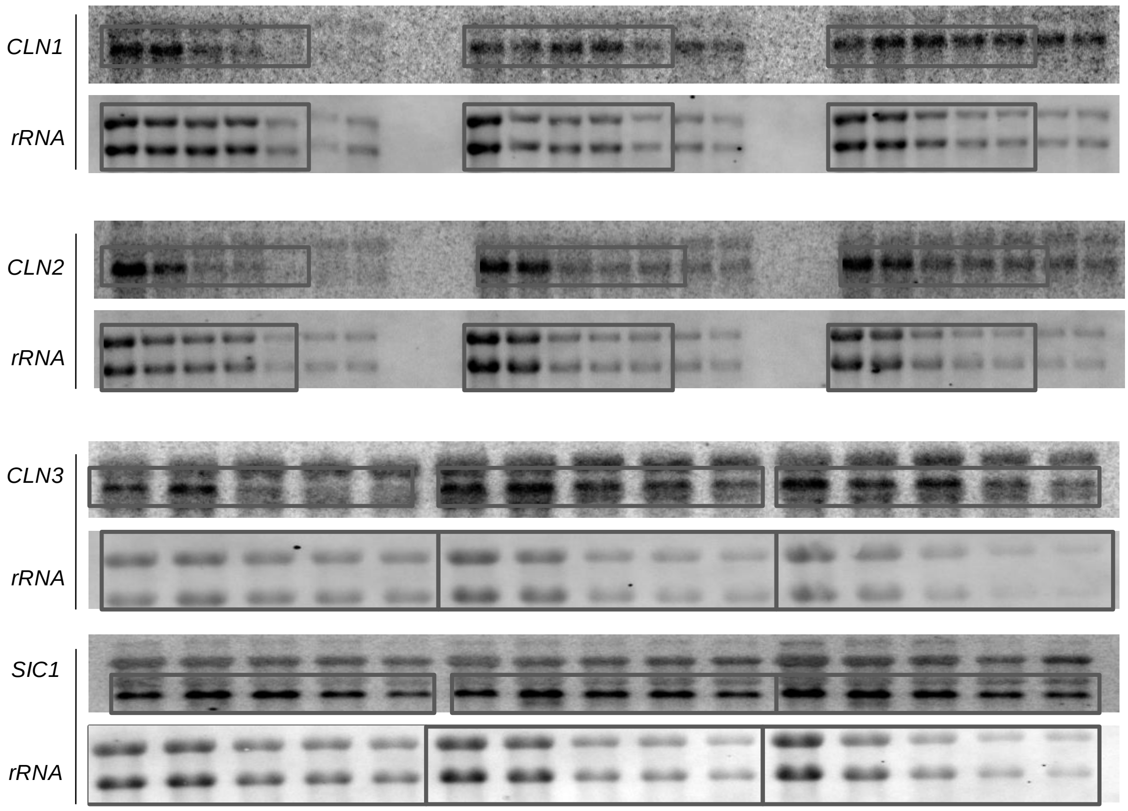

Supplementary Figure 7 | Full-sized scans of Northern blots in Figure 1e.

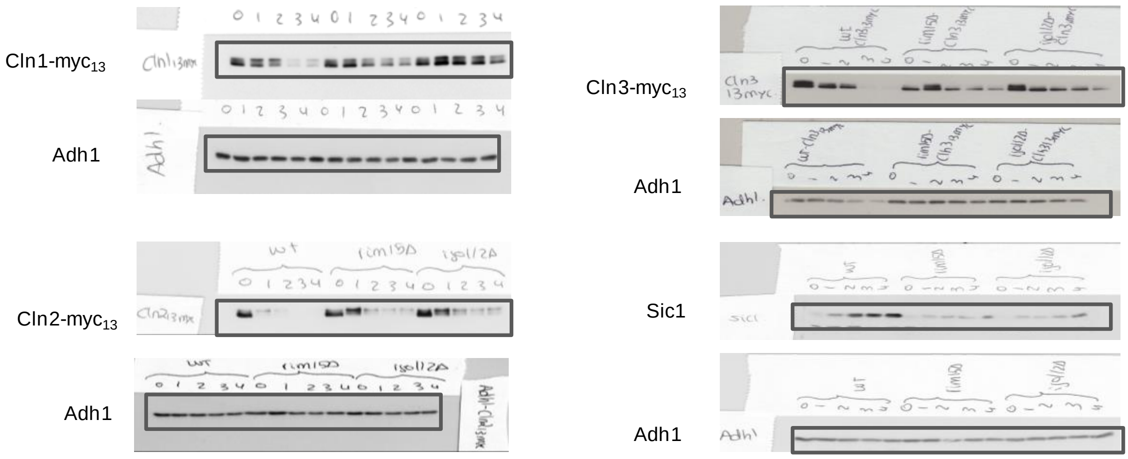

Supplementary Figure 8 | Full-sized scans of Western blots in Figure 1f.

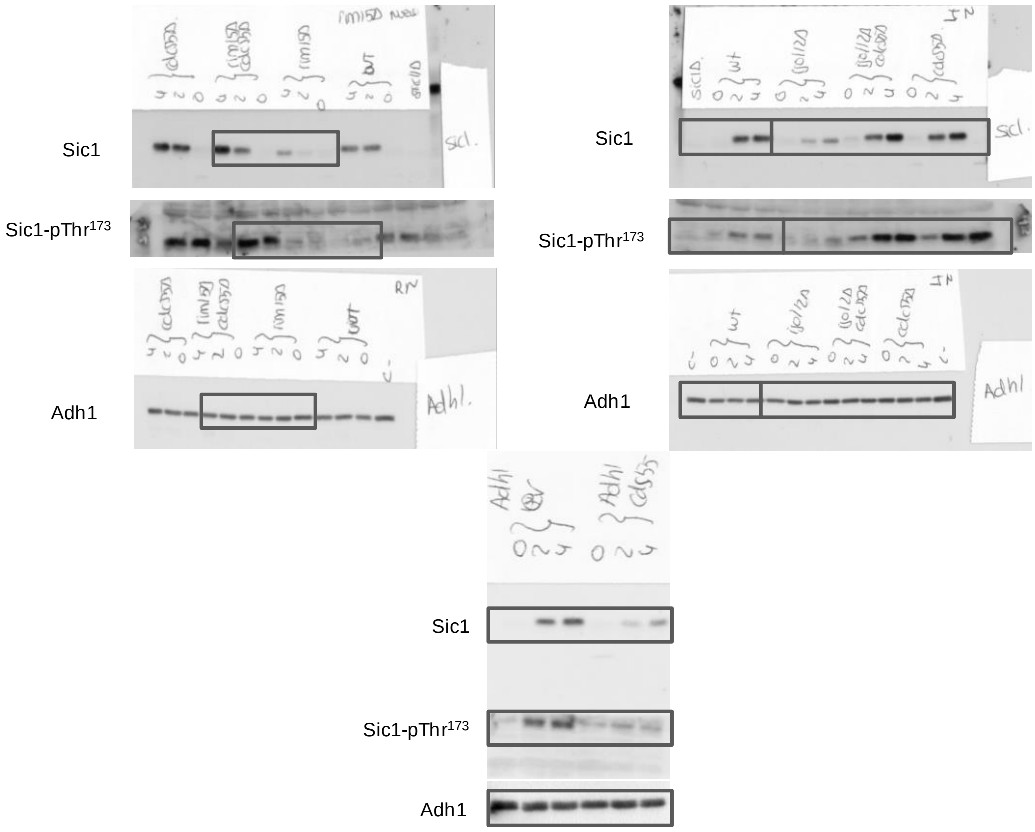

Supplementary Figure 9 | Full-sized scans of Western blots in Figure 2a.

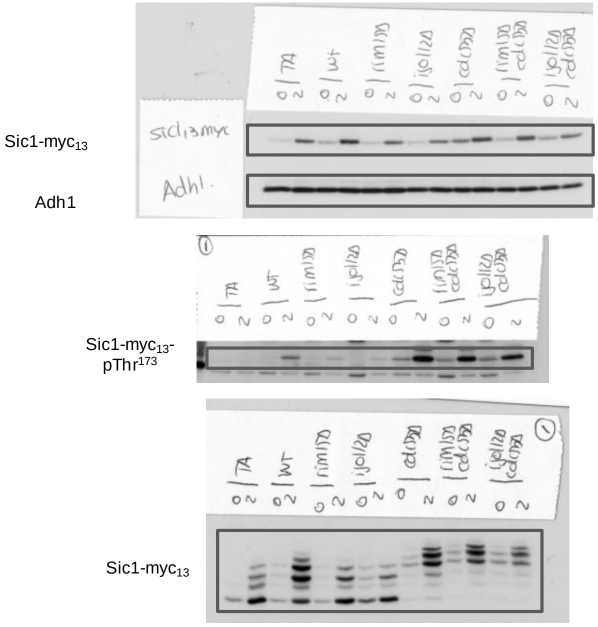

Supplementary Figure 10 | Full-sized scans of Western blots in Figure 2c.

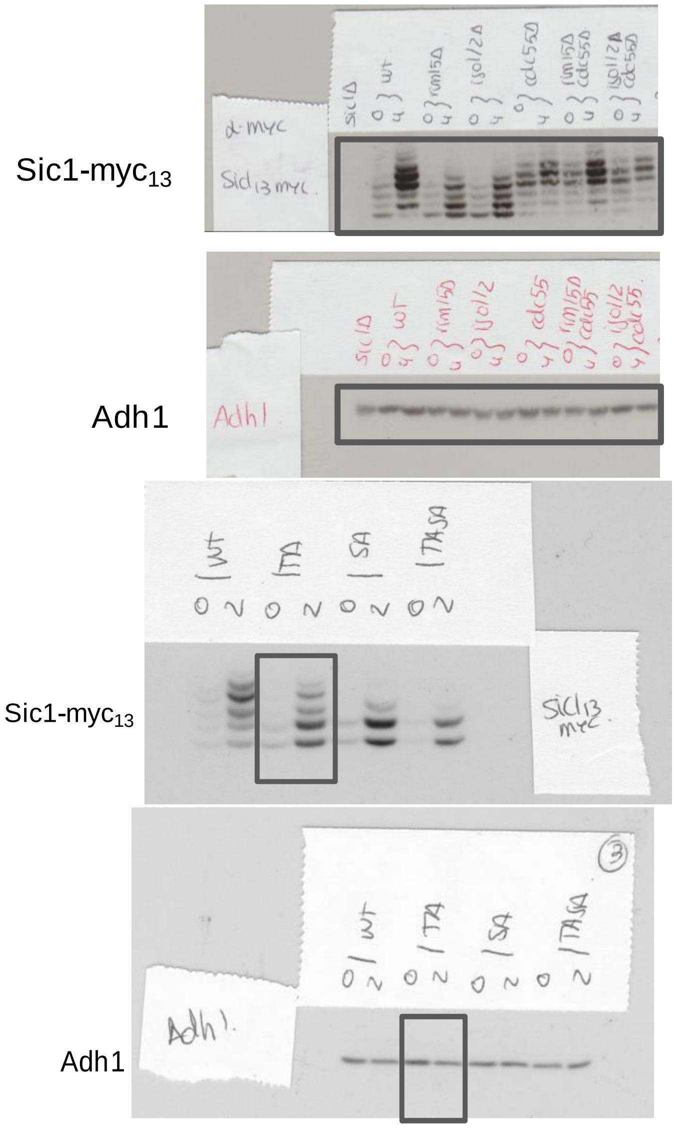

Supplementary Figure 11 |Full-sized scans of Western blots in Figure 2d.

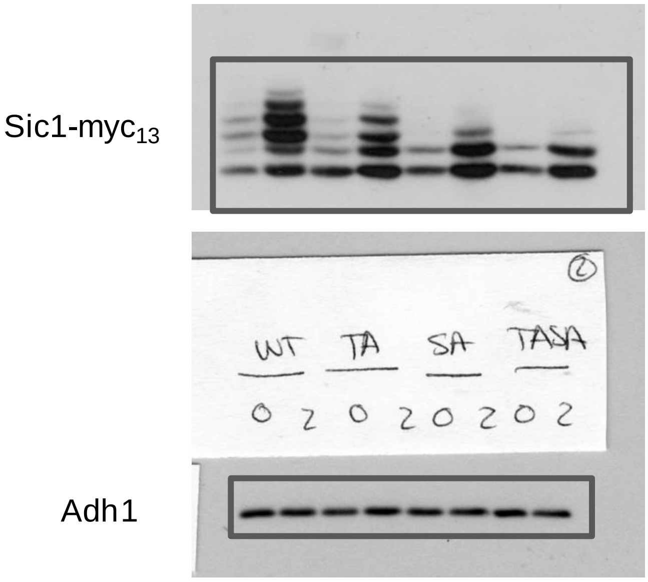

Supplementary Figure 12 | Full-sized scans of Western blots in Figure 2e.

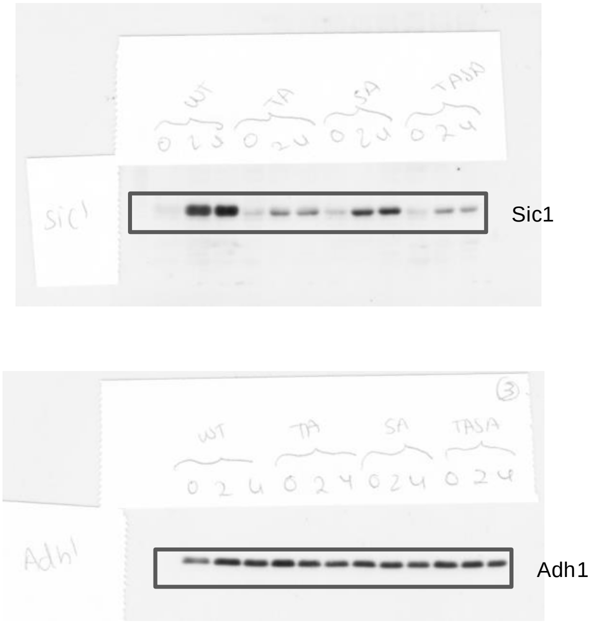

Supplementary Figure 13 | Full-sized scans of Western blots in Figure 2f.

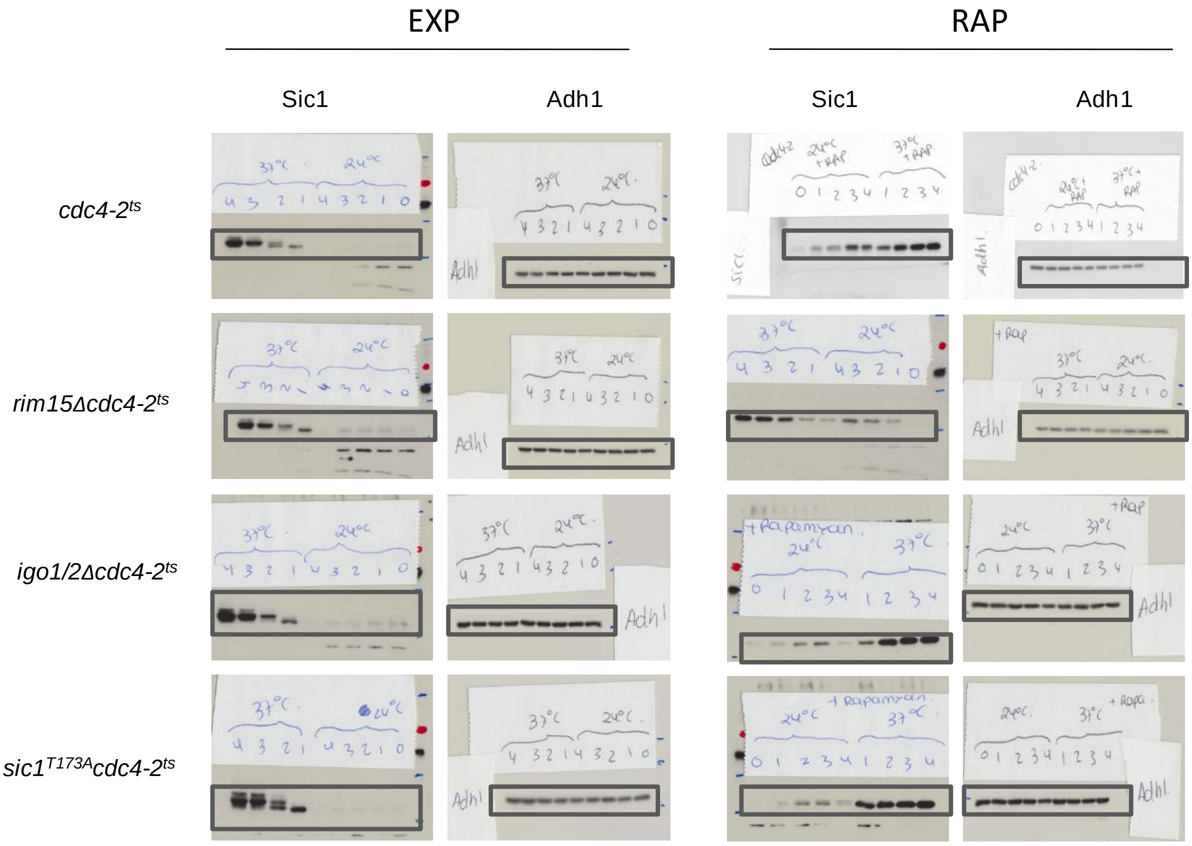

Supplementary Figure 14 | Full-sized scans of Western blots in Figure 3a.

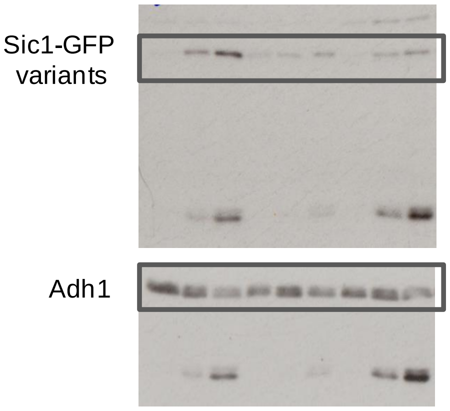

Supplementary Figure 15 | Full-sized scans of Western blots in Figure 3c.

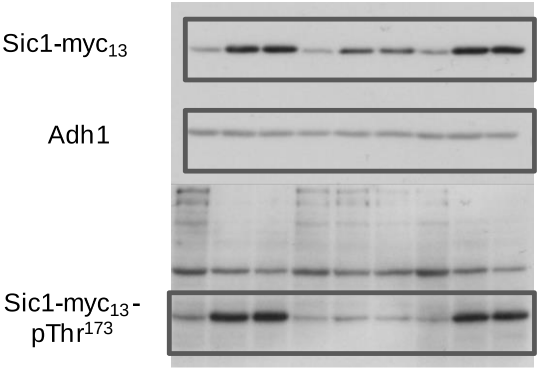

Supplementary Figure 16 | Full-sized scans of Western blots in Figure 4a.

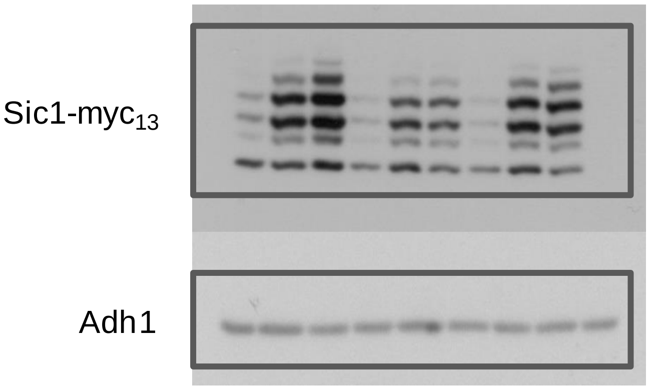

Supplementary Figure 17 | Full-sized scans of Western blots in Figure 4b.

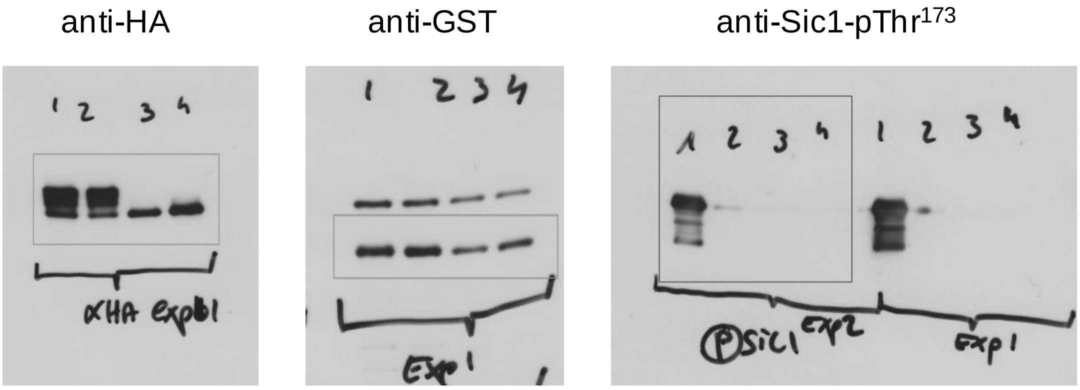

Supplementary Figure 18 | Full-sized scans of Western blots in Figure 4c.

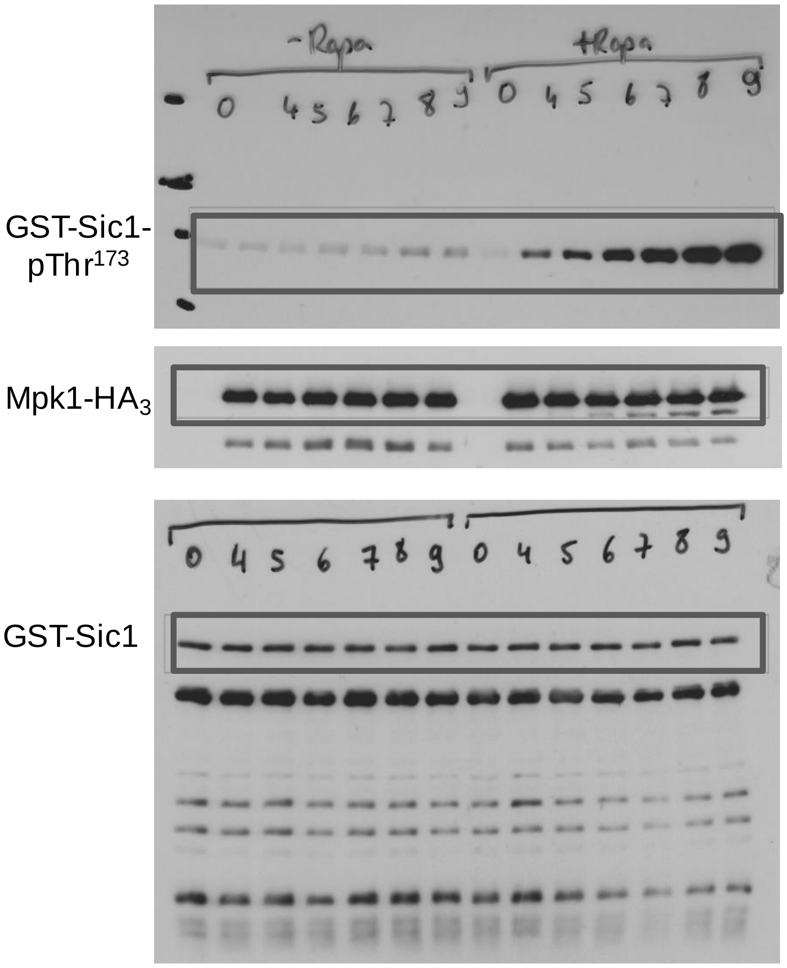

Supplementary Figure 19 | Full-sized scans of Western blots in Figure 4d.

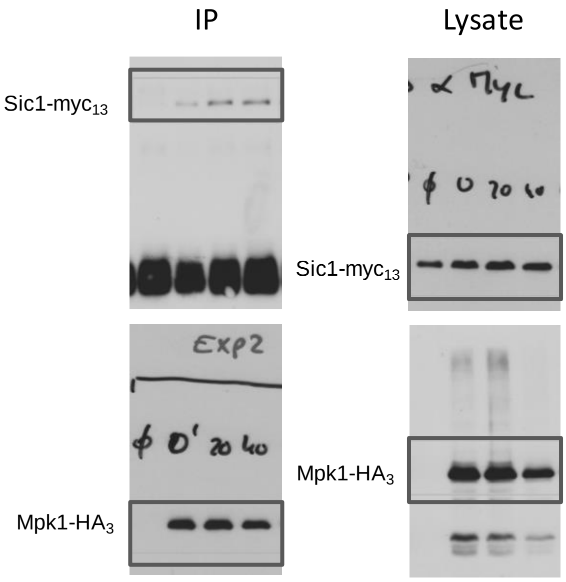

Supplementary Figure 20 | Full-sized scans of Western blots in Figure 4e.

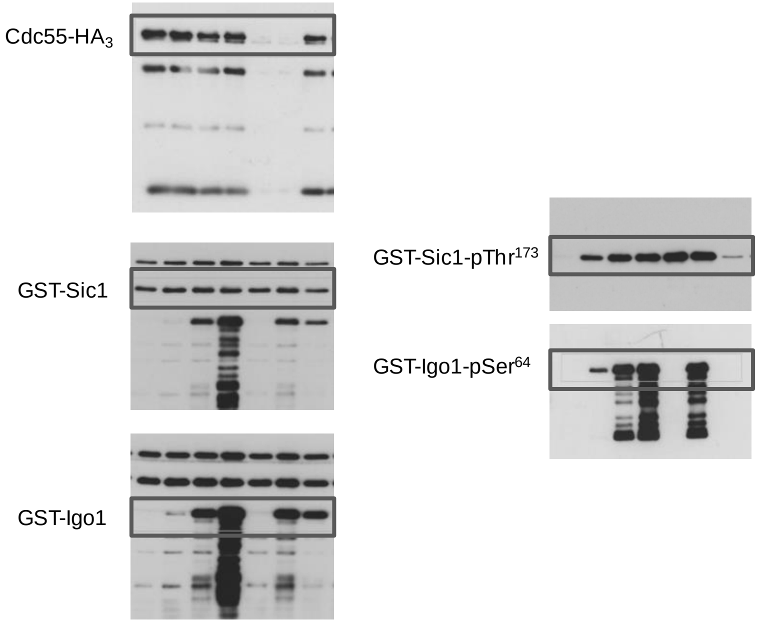

Supplementary Figure 21 | Full-sized scans of Western blots in Figure 5a.

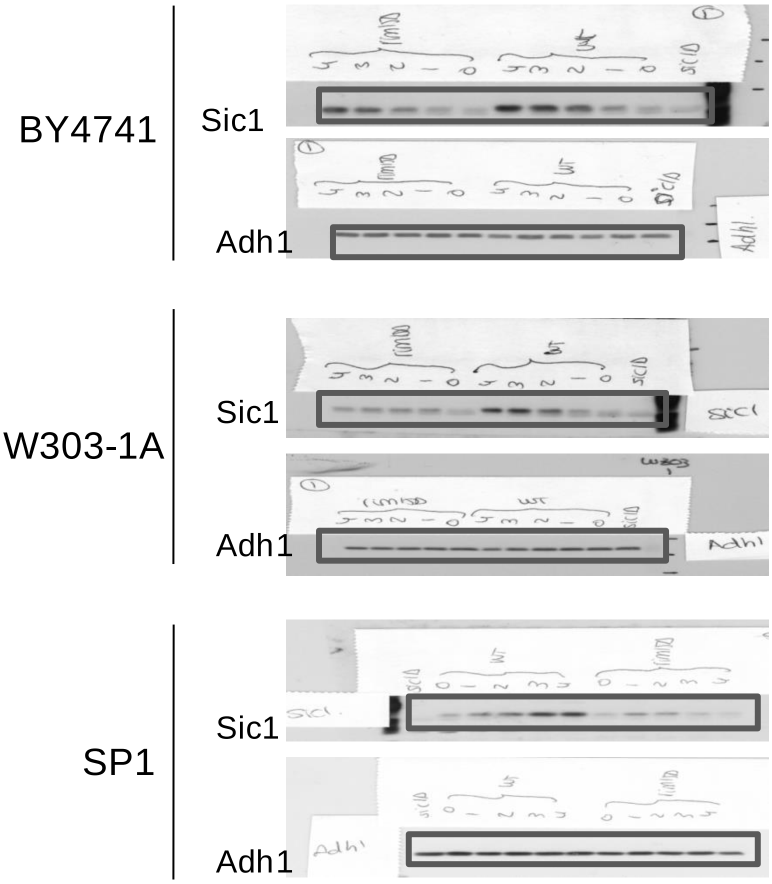

Supplementary Figure 22 | Full-sized scans of Western blots in Supplementary Figure 1.

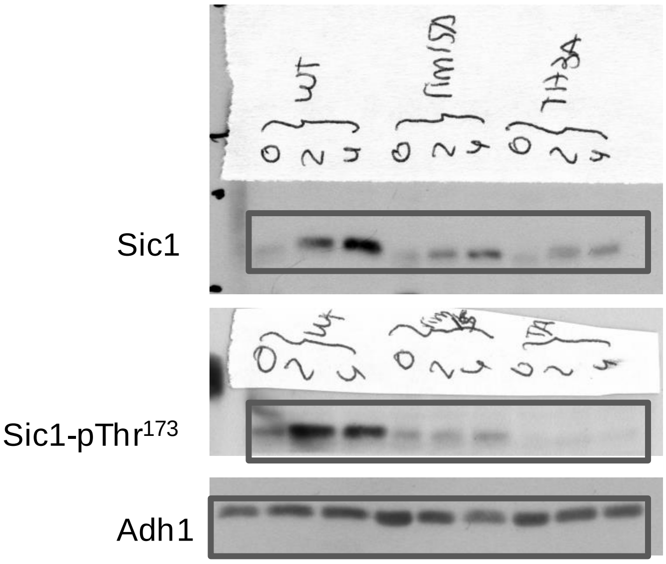

Supplementary Figure 23 | Full-sized scans of Western blots in Supplementary Figure 2.

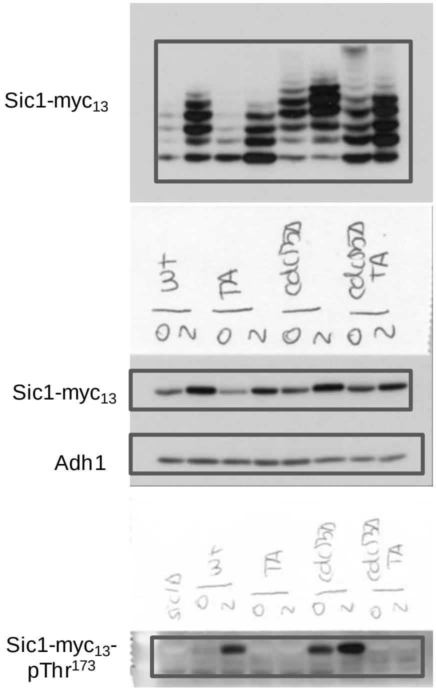

Supplementary Figure 24 | Full-sized scans of Western blots in Supplementary Figure 3.

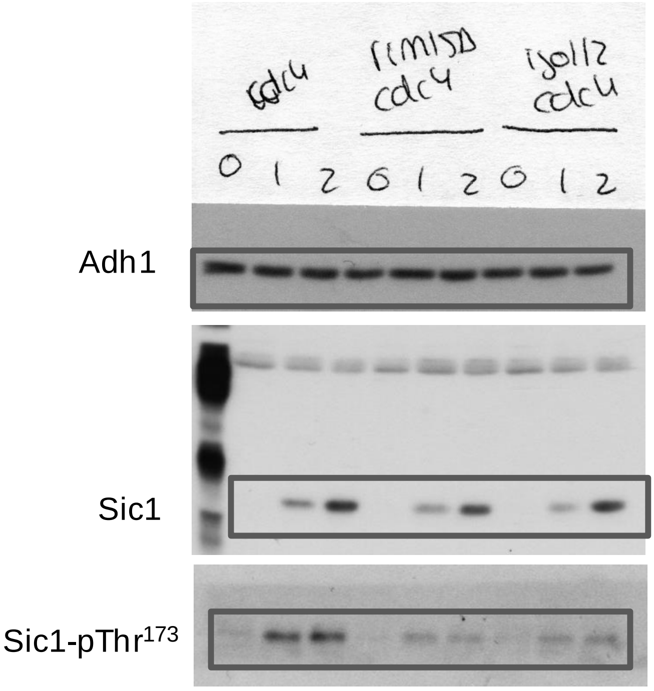

Supplementary Figure 25 | Full-sized scans of Western blots in Supplementary Figure 5.

Supplementary Tables

Supplementary Table 1 | Strains Used in This Study.

Strain	Genotype	Source	Figure
BY4741	*MAT***a***; his3Δ1, leu2Δ0, met15Δ0, ura3Δ0*	reference[1]	1a, S1
W303-1A	*MAT***a***; ade2-1, trp1-1, can1-100, leu2-3,112, his3-11,15, ura3-1*		1a, S1
SP1	*MAT***a***; leu2, his3, trp1, ade8, ura3, can1*	reference[2]	1a, S1
JK9-3D	*MAT***a***; leu2, his4, trp1, ura3, rme1, GAL,HMLa*	reference[3]	1b,1d-f,1i, 2a-b, 2f-g, S2, S4, S6
YMM203	[SP1] *rim15Δ::kanMX*	this study	S1
YSB147	[BY4741] *rim15Δ::natMX, MET15*	reference[5]	S1
CDV95-4A	[W303-1A] *rim15Δ::kanMX*	this study	S1
IP11	[JK9-3D]*rim15Δ::kanMX*	reference[4]	1c-f, 1i, 2a, S2
YMM57-2A	[JK9-3D] *igo1Δ::natMX, igo2Δ::Hph-NT1*	this study	1c-f, 1i, 2a
YMM59	[JK9-3D] *CLN1-myc*$_{13}$*::kanMX*	this study	1f-g
YMM60	[JK9-3D]*CLN2-myc*$_{13}$*::kanMX*	this study	1f-g
YMM61	[JK9-3D] *CLN3-myc*$_{13}$*::kanMX*	this study	1f, 1h
YMM87-2D	[JK9-3D] *rim15Δ::kanMX, CLN1-myc*$_{13}$*::kanMX*	this study	1f-g
YMM88-12B	[JK9-3D] *rim15Δ::kanMX, CLN2-myc*$_{13}$*::kanMX*	this study	1f-g
YMM78-2A	[JK9-3D] *rim15Δ::kanMX, CLN3-myc*$_{13}$*::kanMX*	this study	1f, 1h
YMM85-5D	[JK9-3D] *igo1Δ::natMX, igo2Δ::Hph-NT1, CLN1-myc*$_{13}$*::kanMX*	this study	1f-g
YMM79-9A	[JK9-3D] *igo1Δ::natMX, igo2Δ::Hph-NT1, CLN2-myc*$_{13}$*::kanMX*	this study	1f-g
YMM80-5C	[JK9-3D] *igo1Δ::natMX, igo2Δ::Hph-NT1, CLN3-myc*$_{13:}$*:kanMX*	this study	1f, 1h
YMM55-1C	[JK9-3D] *rim15Δ::kanMX, cdc55Δ::natMX*	this study	2a
YMM90-3D	[JK9-3D] *igo1Δ::natMX, igo2Δ::Hph-NT1, cdc55Δ::natMX*	this study	2a
YMM46	[JK9-3D] *cdc55Δ::natMX*	this study	2a, 5a
YMM98	[JK9-3D] *sic1*^{T173A}*-myc*$_{13}$*::kanMX, EMP46::natMX*	this study	2c-e, S3
YMM143-2A	[JK9-3D] *sic1*^{T173A}*-myc*$_{13}$*::kanMX,cdc55Δ::natMXEMP46::natMX*	this study	S3
YMM63	[JK9-3D] *SIC1-myc*$_{13}$*::kanMX*	this study	2c-e, S3
YMM68-9D	[JK9-3D] *rim15Δ::kanMX, SIC1-myc*$_{13}$*::kanMX*	this study	2c-d
YMM70-6B	[JK9-3D] *igo1Δ::natMX, igo2Δ::Hph-NT1, SIC1-myc*$_{13}$*::kanMX*	this study	2c-d
YMM69-1C	[JK9-3D] *cdc55Δ::natMX, SIC1-myc*$_{13}$*::kanMX*	this study	2c-d, S3
YMM100-9D	[JK9-3D] *rim15Δ::kanMX, cdc55Δ::natMX, SIC1-myc*$_{13}$*::kanMX*	this study	2c-d
YMM96	[JK9-3D] *igo1Δ::natMX, igo2Δ::Hph-NT1, cdc55Δ::natMX, SIC1-myc*$_{13}$*::kanMX*	this study	2c-d
YMM91	[JK9-3D] *sic1*^{T173A}*, EMP46::natMX*	this study	2f-g, S2, S4
YMM101	[JK9-3D] *sic1*^{S191A}*, EMP46::natMX*	this study	2f-g, S4
YMM103	[JK9-3D] *sic1*$^{T173A/S191A}$*, EMP46::natMX*	this study	2f-g, S4
YMM105	[JK9-3D] *sic1*^{S191A}*-myc13::kanMX, EMP46::natMX*	this study	2e
YMM133	[JK9-3D] *sic1*$^{T173A/S191A}$*-myc13::kanMX, EMP46::natMX*	this study	2e
YMM114	[JK9-3D] *cdc4-2::kanMX*	this study	3a-b, S5
YMM117-3A	[JK9-3D] *rim15Δ::kanMX, cdc4-2::kanMX*	this study	3a-b, S5
YMM116-4A	[JK9-3D] *igo1Δ::natMX, igo2Δ::Hph-NT1, cdc4-2::kanMX*	this study	3a-b, S5
YMM118-2D	[JK9-3D] *sic1*^{T173A}*, EMP46::natMX, cdc4-2::kanMX*	this study	3a-b
YMM77	[JK9-3D] *SIC1-GFP(S65T)::kanMX*	this study	3c-d
YMM97-7B	[JK9-3D] *rim15Δ::kanMX, SIC1-GFP(S65T)::kanMX*	this study	3c
YMM99	[JK9-3D] *sic1*^{T173A}*-GFP(S65T)::kanMX*	this study	3c-d
YMM67-1C	[JK9-3D] *sic1Δ::kanMX*	this study	2a
YMM53	[JK9-3D] *mpk1Δ::kanMX*	this study	4c-f, S6
YMM65-2D	[JK9-3D] *mpk1Δ::kanMX, SIC1-myc*$_{13}$*::kanMX*	this study	4a-b
YMM204-14C	[JK9-3D] *hog1Δ::kanMX, SIC1-myc*$_{13}$*::kanMX*	this study	4a-b
YMM64-3C	[JK9-3D] *rim15Δ::kanMX, mpk1Δ::kanMX*	this study	4f
YMM111-2A	[JK9-3D] *igo1Δ::natMX, igo2Δ::Hph-NT1, mpk1Δ::kanMX*	this study	4f
YMM130	[JK9-3D] *hog1Δ::kanMX*	this study	S6

Supplementary Table 2 | Plasmids Used in This Study.

Plasmid	Genotype	Source	Figure
pRS416	*CEN/ARS*, *URA3*	reference[1]	1b
pMM5	[pRS416] *RME1*	this study	1b
p1308	[pRS414]	reference[1]	1d, 2d
p1309	[pRS415]	reference[1]	1d, 2d
p1310	[pRS416]	reference[1]	1d, 2d
pMM10	[pRS415]*HIS4*	this study	1d, 2d
pSB004	[pRS416] *ADH1*p-*CDC55*	reference[5]	2a-b
p834	[pRS416] *ADH1*p	reference[6]	2a-b
pMM6	[pRS416]*MPK1-HA*$_3$	this study	4c-e
pMM7	[pRS416]*mpk1*^{K54R}-*HA*$_3$	this study	4c
pMM8	pGEX-*SIC1*	this study	4c-d, 5a
pMM9	pGEX-*sic1*^{T173A}	this study	4c
pMJA2610	[pRS416]*CDC55-HA*$_3$	this study	5a
pCDV487	pHAC195-*GAL1-GST-RIM15,* 2 µ,*URA3*	reference[4]	5a
pLC1092	pGEX-*IGO1*	reference[7]	5a
pLC1134	pGEX-*igo1*^{S64A}	reference[7]	5a

Supplementary References

1. Brachmann, C.B. *et al.* Designer deletion strains derived from *Saccharomyces cerevisiae* S288C: a useful set of strains and plasmids for PCR-mediated gene disruption and other applications. *Yeast* **14**, 115-132 (1998).
2. Toda, T. *et al.* In yeast, RAS proteins are controlling elements of adenylate cyclase. *Cell* **40**, 27-36 (1985).
3. Heitman, J., Movva, N.R. & Hall, M.N. Targets for cell cycle arrest by the immunosuppressant rapamycin in yeast. *Science* **253**, 905-909 (1991).
4. Pedruzzi, I. *et al.* TOR and PKA signaling pathways converge on the protein kinase Rim15 to control entry into G_0. *Mol. Cell* **12**, 1607-1613 (2003).
5. Bontron, S. *et al.* Yeast endosulfines control entry into quiescence and chronological life span by inhibiting protein phosphatase 2A. *Cell Rep* **3**, 16-22 (2013).
6. Mumberg, D., Müller, R. & Funk, M. Yeast vectors for the controlled expression of heterologous proteins in differentgenetic backgrounds. *Gene* **156**, 119-122 (1995).
7. Talarek, N. *et al.* Initiation of the TORC1-regulated G_0 program requires Igo1/2, which license specific mRNAs to evade degradation via the 5'-3' mRNA decay pathway. *Mol Cell* **38**, 345-355 (2010).
